# Mitochondrial genome characteristics and phylogenetic analysis of the medicinal and edible plant *Mesona chinensis* Benth

**DOI:** 10.3389/fgene.2022.1056389

**Published:** 2023-01-12

**Authors:** Danfeng Tang, Suhua Huang, Changqian Quan, Yuan Huang, Jianhua Miao, Fan Wei

**Affiliations:** ^1^ Guangxi Key Laboratory of Medicinal Resources Protection and Genetic Improvement, Guangxi Botanical Garden of Medicinal Plants, Nanning, China; ^2^ Guangxi Engineering Research Center of TCM Resource Intelligent Creation, Guangxi Botanical Garden of Medicinal Plants, Nanning, China; ^3^ College of Pharmacy, Guangxi Medical University, Nanning, China

**Keywords:** *Mesona chinensis* Benth, mitogenome, characteristics, RSCU, KaKs, constitution analysis, phylogenetic analysis

## Abstract

*Mesona chinensis* Benth (MCB) (or Platostoma palustre or Platostoma chinense) is an important edible and medicinal plant in China. However, the mitochondrial genome (mitogenome, or mtDNA) of MCB has not been characterized or reported yet. In this study, we first sequenced and characterized the complete mitogenome of MCB. The MCB mitogenome was 494,599 bp in length and encoded 59 genes containing 37 protein-coding genes (PCGs), 19 tRNAs, and 3 rRNAs. Gene transfer analysis revealed that a total of 12 transfer segments with more than 93% identity (total length of 25,427 bp) were detected in the MCB mitogenome. Simple sequence repeats (SSR) analysis showed that 212 simple sequence repeats (SSR) were identified. Repeat sequence analysis revealed 305 repeat sequences (158 forward and 147 palindromic repeats) ranging from 30 bp to 48,383 bp and the 30–39 bp repeats were the majority type. Relative synonymous codon usage (RSCU) analysis uncovered that in total, 9,947 codons were encoding the protein-coding genes (PCGs). Serine (909, 9.1%) and leucine (879, 8.8%) were the two most abundant amino acids, while terminator (32, .3%) was the least abundant amino acid. Ka/Ks analysis indicated that almost all genes were subject to purification selection, except *ccmB*. Analysis of Lamiaceae mitogenomes constitution revealed that *atpB* and *atpE* were unique to the *Rotheca serrata* and *Salvia miltiorrhiza* mitogenomes. *mttB* gene loss was unique to the *Boea hygrometrica* mitogenome. The core fragments of the Lamiaceae mitogenomes harbored a higher GC content than the specific and variable fragments. In addition, phylogenetic analysis revealed that MCB was closely related to *Salvia miltiorrhiza* based on the mitogenomes. The current study provided valuable genomic resources for understanding and utilizing this important medicinal plant in the future.

## Introduction

Mitochondria, semi-autonomous organelles in eukaryotic cells, have relatively independent genetic systems (Mitochondrial DNA, mtDNA) (Gray, 2012; Chen et al., 2017) and have always been a hot topic in the field of molecular biology. Mitogenome is widely used in system and evolutionary biology, population genetics, and conservation biology (Zhong et al., 2020). The mitogenomes of higher plants vary dramatically in size (Backert et al., 1997), even among species of the same genus, ranging from 66 Kbp of *Viscum scurruloideum* (Skippington et al., 2015) to 11.3 Mbp of *Silene conica* (Sloan et al., 2012). And the number of genes in plant mitogenomes ranges from 19 (*Viscum album*) to 221 (*Capsicum annuum*, Solanaceae) (Jo et al., 2011; Petersen et al., 2015). Despite plant mitogenomes differ in size and number of genes, they encode roughly the same gene products, including rRNAs, tRNAs, and protein subunits required for respiratory chain complexes (Li et al., 2011; Bi et al., 2020). Plant mitogenomes are usually large in comparison with animal or fungal mitogenomes and contain fewer genes than their plastid counterpart and are also complex due to the presence of a large number of non-coding regions and the introgression of foreign DNA from the nuclear or chloroplast genome (Alverson et al., 2010). Plant mitogenome is characterized by dramatic and rapid structural changes, which makes the conventional modes of sequencing and assembly less effective. Currently, there are two strategies to obtain a complete plant mitogenome. One is to separately isolate mitochondria first, then extract mitochondrial DNA and sequence the mitochondrial DNA (Wang et al., 2008). The other one is to directly extract the total genomic DNA without isolation and purification of mitochondria and then screen out the mitochondrial genome for assembly based on a sequencing library (Férandon et al., 2013; Song et al., 2019). As the assembly techniques improve and the cost of sequencing reduces, the number of plant mitogenomes should raise rapidly (Yuan et al., 2016).

*Mesona chinensis* Benth (MCB) is a traditional Chinese herbal medicine, which has been used for thousands of years in China, India, Indonesia, Malaysia, Philippines, Thailand, etc (Lin et al., 2018). MCB is an annual herbaceous plant and distributes in South China and Southeast Asian countries (Huang et al., 2019; Ren et al., 2019; Tang et al., 2020). In China, it is one of the most important medicinal and edible plants (Tang et al., 2022a). MCB belongs to the Lamiaceae family, which is the largest family-level clade in the Lamiales and consists of more than 7,000 species of 236 genera globally (Li et al., 2016; Tang et al., 2022b). However, compared with a large number of Lamiaceae species, the number of mitogenomes sequenced within this family is still minimal. To date, the mitogenomes of Lamiaceae plants that have been released and uploaded to NCBI (National Center for Biotechnology Information) only include *Ajuga reptans*, *Dracocephalum moldavica*, *R. serrata*, *S. miltiorrhiza*, *S. splendens*, etc. Therefore, it is necessary to obtain the MCB mitogenome to enrich the database of plant mitogenome. In addition, in recent years, although there have been more and more research reports on MCB, suggesting that it is getting more and more attention, the research foundation of MCB still needs to be further strengthened, especially the data on the molecular biology of this species need to be further explored.

Hence, in this study, we sequenced and characterized the complete mitogenome of MCB using the Next-Generation Sequencing and Nanopore technologies, and performed the phylogenetic analysis based on mtDNA. To the best of our knowledge, this is the first assembly of the MCB mitogenome, which provides valuable information for the molecular biology research of this species and also enriches the database of plant mitogenome.

## Materials and methods

### DNA sequencing and genome assembly

*M. chinensis* Benth (MCB) was planted in Guangxi Botanical Garden of Medicinal Plants (N 22°51′, E 108°19′), Nanning city, China. Total genomic DNA isolation was carried out using a modified cetyltrimethylammonium bromide (CTAB) (Allen et al., 2006) method and applied to 500 bp paired-end library construction using the NEBNext^®^ Ultra^™^ DNA Library Prep Kit for Illumina sequencing. Sequencing was performed on the Illumina NovaSeq 6,000 platform. About 4.1 Gb of raw data from MCB were produced with 150 bp paired-end read lengths. For Oxford Nanopore library construction and sequencing, purified DNA was sheared to 20-kb fragments using a Covaris g-tube (Covaris) and purified with AMPure beads. The DNA concentration was measured with a Qubit fluorometer and end-repaired using the NEBNext End Repair Module. The final library was sequenced using the MinION device.

GetOrganelle v1.6.4 (Jin et al., 2020) was employed for *de novo* assembly using the mitogenome of the closely related species *Salvia miltiorrhiza* (GenBank Acc. No. KF177345.1) as the seed sequence. We extracted the potential mitochondrion reads from the pool of illumina reads using BLAST searches against the mitogenome of *S. miltiorrhiza* and the GetOrganelle results. Then the mitochondrial illumina reads were obtained to perform mitogenome *de novo* assembly using the SPAdes-3.14.0 (Antipov et al., 2016) package, which generated ∼50 contigs of mitogenome.

Clean Nanopore long-reads were aligned against the GetOrganelle and SPAdes assembled scaffolds using the BWA men program. All aligned Nanopore reads were extracted to perform self-correction and mitogenome *de novo* assembly using the Canu v2.0 (Koren et al., 2017) package, followed by error correction using the racon v1.4.3 (Fang et al., 2020) and pilon v1.21. The Nanopore assembly sequences were aligned against a nucleotide sequence database to remove the chloroplast and contaminating sequences and then were checked if the sequences have overlaps and connections between them (Li et al., 2021). Then the retained mitochondrial long contigs were connected into a ring. Finally, the complete MCB mitogenome was obtained after polishing with both Nanopore long reads and Illumina short reads.

### Genome annotation

The online GeSeq tool with default parameters was used to predict the protein-coding genes, transfer RNA (tRNA) genes, and ribosome RNA (rRNA) genes (Tillich et al., 2017; Tang et al., 2021b). The position of each coding gene was determined by BLAST (Altschul et al., 1990) searches against the reference mitochondrial genes. Manual corrections of genes for start/stop codons and intron/exon boundaries were conducted in SnapGene Viewer by comparing them with the reference genome. The OrganellarGenomeDRAW (https://ogdraw.mpimp-golm.mpg.de/cgi-bin/ogdraw.pl) (Greiner et al., 2019) tool was used to draw the mitochondrial genome map of MCB. Functional annotations were conducted using sequence-similarity blast searches with a typical cut-off E-value of 10^−5^ against several publicly available protein databases: Swiss-Prot, NCBI non-redundant (Nr) protein database, Clusters of Orthologous Groups (COGs), Kyoto Encyclopedia of Genes and Genomes (KEGG), and Gene Ontology (GO) terms (Fang et al., 2020).

### Gene transfer between the mitogenome and chloroplast genome

The MCB mitogenome was compared with its chloroplast genome (Tang et al., 2021b) by BLASTn and the selected parameter E value was less than 1e^−5^.

### Repeat sequence analysis

REPuter (Kurtz et al., 2001) (https://bibiserv.techpak.uni-bielefeld.de/computer/) software was used for repeat sequence analysis. The MISA-MIcroSAtellite identification tool (Beier et al., 2017) (https://pgrc.ipk-gatersleben.de/misa/) was employed to identify the SSR sequences.

### Collinearity analysis

The genome alignment between the assembly sequences and other sequences was performed using nucmer (4.0.0beta2) software with the --maxpatch parameter to generate dot plot. The BLASTN (2.10.1 +) software (-word_size is 7, E-value is 1e^−5^, and the fragments with alignment length greater than 300 bp are screened) was used to sequentially compare the assembled species with the selected species and draw a collinearity map.

### Relative synonymous codon usage analysis

RSCU refers to the actual usage frequency of a codon divided by the theoretical usage frequency of the codon. The unique coding sequences (CDS) were filtered and calculated using a Perl script written by ourselves.

### KaKs analysis

The mafft v7.310 (Katoh and Standley, 2013) (https://mafft.cbrc.jp/alignment/software/) software was used for gene sequence alignment. The KaKs_Calculator v2.0 (https://sourceforge.net/projects/kakscalculator2/) software was employed to calculate the KaKs value of the gene with the MLWL calculation method.

### Lamiaceae mitogenomes constitution analysis

To study the constitution variation of the Lamiaceae mitogenomes, we performed multiple whole genome alignment of the mitogenomes using Mugsy (Angiuoli and Salzberg, 2011). The Lamiaceae mitogenomes were parsed to three different types of fragments including core fragments, specific fragments, and variable fragments from the output of Mugsy using the customized shell scripts. In this study, the core fragments were shared by all 9 Lamiaceae mitogenomes and the variable fragments were shared by a few mitogenomes. And the specific fragments were unique to only one mitogenome. We obtained the size accumulation curves of the pan mitogenome and core mitogenome of 9 Lamiaceae species using PanGP v1.0.1 (Zhao et al., 2014) as well as multiple whole genome alignment analysis by random sampling of up to 500 replicates for each group. The size information of core mitogenome was the accumulation of the sizes of the core fragments of each group and the size information of pan mitogenome was the accumulation of the sizes of specific fragments, variable fragments, and core fragments, of which the sizes of homologous sequences among the core and variable fragments calculated once.

### Phylogenetic analysis

To determine the phylogenetic position of MCB, we constructed the phylogenetic tree based on the whole mitogenomes of 41 species. The GenBank accession numbers of all the studied species were listed in Supplementary Table S1. The PhyML V3.0 software (https://www.atgc-montpellier.fr/phyml/) was employed for phylogenetic analysis by maximum likelihood (ML) method, Bayes correction, and 1,000 bootstrap replicates to calculate bootstrap values (Guindon et al., 2010).

In addition, a total of 24 protein-coding genes were selected for phylogenetic analysis. Alignment of conservative genes was performed with MUSCLE (codons), and then Gblocks_0.91b (Gblocks: https://www.plob.org/article/7933.html) was used for extraction of conservative regions. jModelTest was employed to evaluate the optimal nucleotide substitution model of each gene, and the evaluation result of Akaike information criterion (AIC) shall prevail. The gene tree was constructed with PhyML.

## Results

### MCB mitogenome sequencing and assembly

In this study, the MCB mitogenome was sequenced by Illumina and Nanopore sequencing platforms. Overall, 4,136,459,100 raw data (Q30 = 88.88%) and 3,487,825,086 bp clean data (Q30 = 91.65%) were obtained using the Illumina platform (Supplementary Table S2) (SRA, SRR21196490). Regarding the Nanopore sequencing, a total of 72,273,957 bases were generated and the total reads number was 9,458 (SRA, SRR21196489). The subreads with N50 and N90 were 8,697 bp and 4,741 bp, respectively. The largest length of subreads was 65,388 bp (Supplementary Table S3). Through *de novo* assembling, the MCB mitogenome sequence was obtained and deposited in GenBank with accession number OP537517.

**FIGURE 1:**
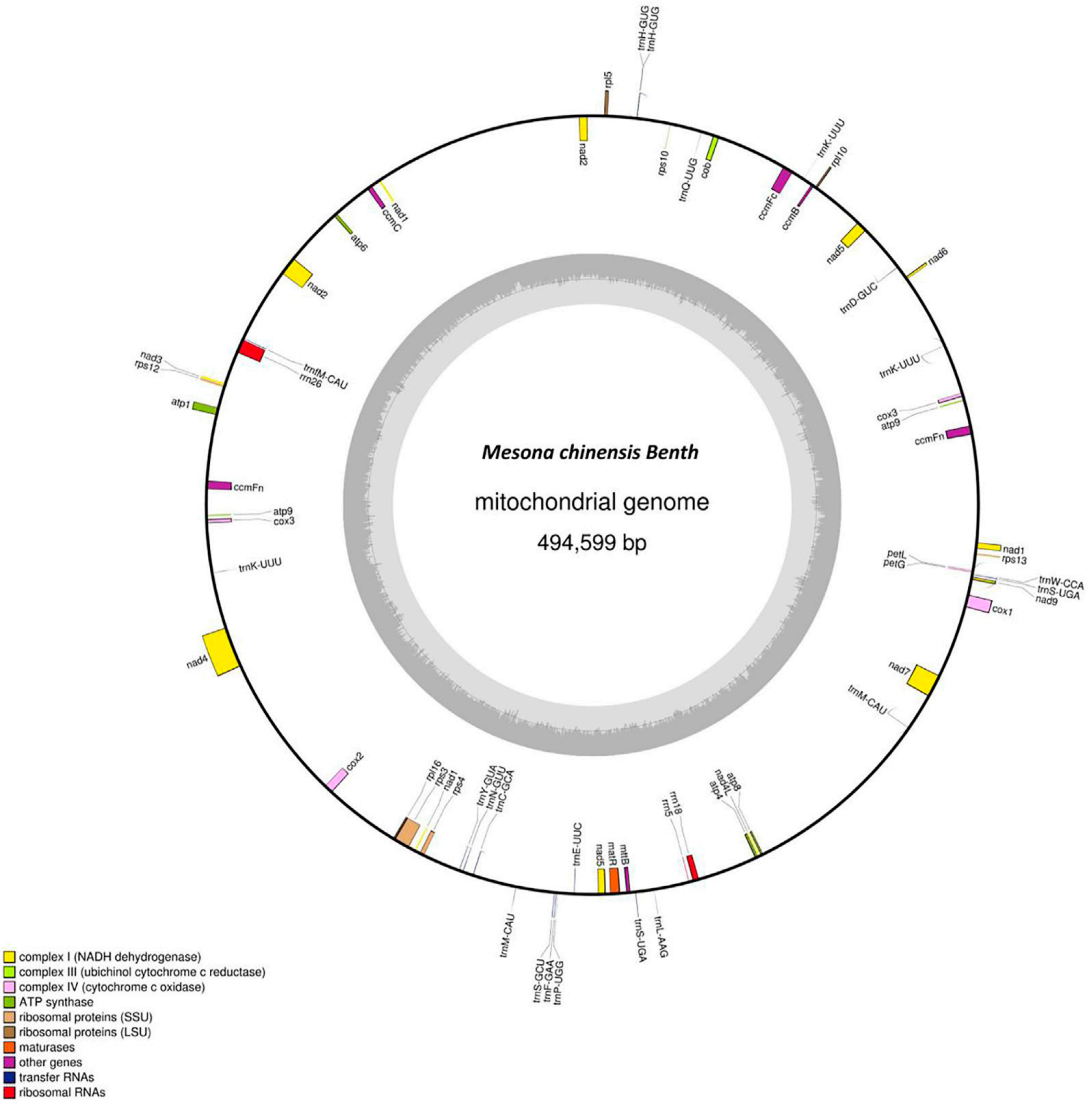
The circular mitochondrial genome of *Mesona chinensis* Benth (MCB). Genes shown outside of the circle are transcribed clockwise, whereas genes on the inside are transcribed counterclockwise. Genes belonging to different functional groups are color-coded. GC content is represented on the inner circle by the dark gray plot.

The complete mitogenome of MCB was 494,599 bp in length (Figure 1). The MCB mitogenome organization and base composition were summarized in Table 1. The total GC content of the MCB mitogenome was 44.21%, while the gene’s GC content and intergenic’s GC content were 42.73% and 44.32%, respectively. The gene total length was 33,166 bp, while the intergenic region length was 461,433 bp.

**TABLE 1:** Assembly statistics for the *Mesona chinensis* Benth mt genome.

Item	*Mesona chinensis Benth*
Genome Size (bp)	494,599
GC Content (%)	44.21
Gene Number	37
Gene Total Length (bp)	33,166
Gene Average Length (bp)	896
Gene’s GC Content	42.73
% of Genome (Genes)	6.71
Intergenic Region Length (bp)	461,433
Intergenic’s GC Content	44.32
% of Genome (Intergenic)	93.29

The mitogenome of MCB encoded 59 genes containing 37 PCGs, 19 tRNAs, and 3 rRNAs (Table 2; Supplementary Table S4). Among these, three PCGs (*cox3*, *atp9*, *ccmFn*) and two tRNAs (*trnK-UUU* and *trnM-CAU*) contained two copies, while the remaining genes had one copy. Three ribosomal subunits (*rrn18*, *rrn5*, and *rrn26*) were encoded with lengths of 1,282 bp, 112 bp, and 2,875 bp, respectively (Supplementary Table S4). Of the PCGs, *nad1*, *nad2*, *nad4*, *nad5*, *nad7*, *cox1*, *ccmFc*, *rps3*, and *matR* contained introns (Supplementary Table S5). *nad2* included four introns, while *cox1*, *ccmFc*, *rps3*, and *matR* had only one intron and the other four genes contained three introns. As for tRNAs, the length of all 19 tRNAs was 1,433 bp (Supplementary Table S4) and the length of each tRNA ranged from 65 bp to 90 bp (Supplementary Table S6).

**TABLE 2:** Gene content of MCB mt genome.

Group of genes	Names of genes
Complex I (NADH dehydrogenase)	nad1*, nad2*, *nad3*, nad4*, *nad4L*, nad5*, *nad6*, nad7*, *nad9*
Complex III (ubiquinol cytochrome c reductase)	*cob*
Complex IV (cytochrome c oxidase)	cox1*, *cox2*, *cox3* (×2)
Complex V (ATP synthase)	*atp1*, *atp4*, *atp6*, *atp8*, *atp9* (*×*2)
Cytochrome c biogenesis	ccmC, ccmFc*, ccmFn (*×2*), *ccmB*
Ribosomal proteins (SSU)	rps3*, rps4, rps12, rps13
Ribosomal proteins (LSU)	rpl5, rpl10
Maturases	matR*
Transport membrane protein	*mttB*
Subunits of cytochrome	petG, petL
Pseudogene	rps10, rpl16
Ribosomal RNAs	*rrn5*, *rrn18*, *rrn26*
Transfer RNAs	trnK-UUU(×2), trnD-GUC, trnQ-UUG, trnH-GUG, trnfM-CAU, trnY-GUA, trnN-GUU, trnC-GCA, trnM-CAU(×2), trnS-GCU, trnF-GAA, trnP-UGG, trnE-UUC, trnS-UGA, trnL-AAG, trnS-UGA, trnW-CCA

*Genes contain introns.

### Gene transfer between the mitogenome and chloroplast genome of MCB

**FIGURE 2:**
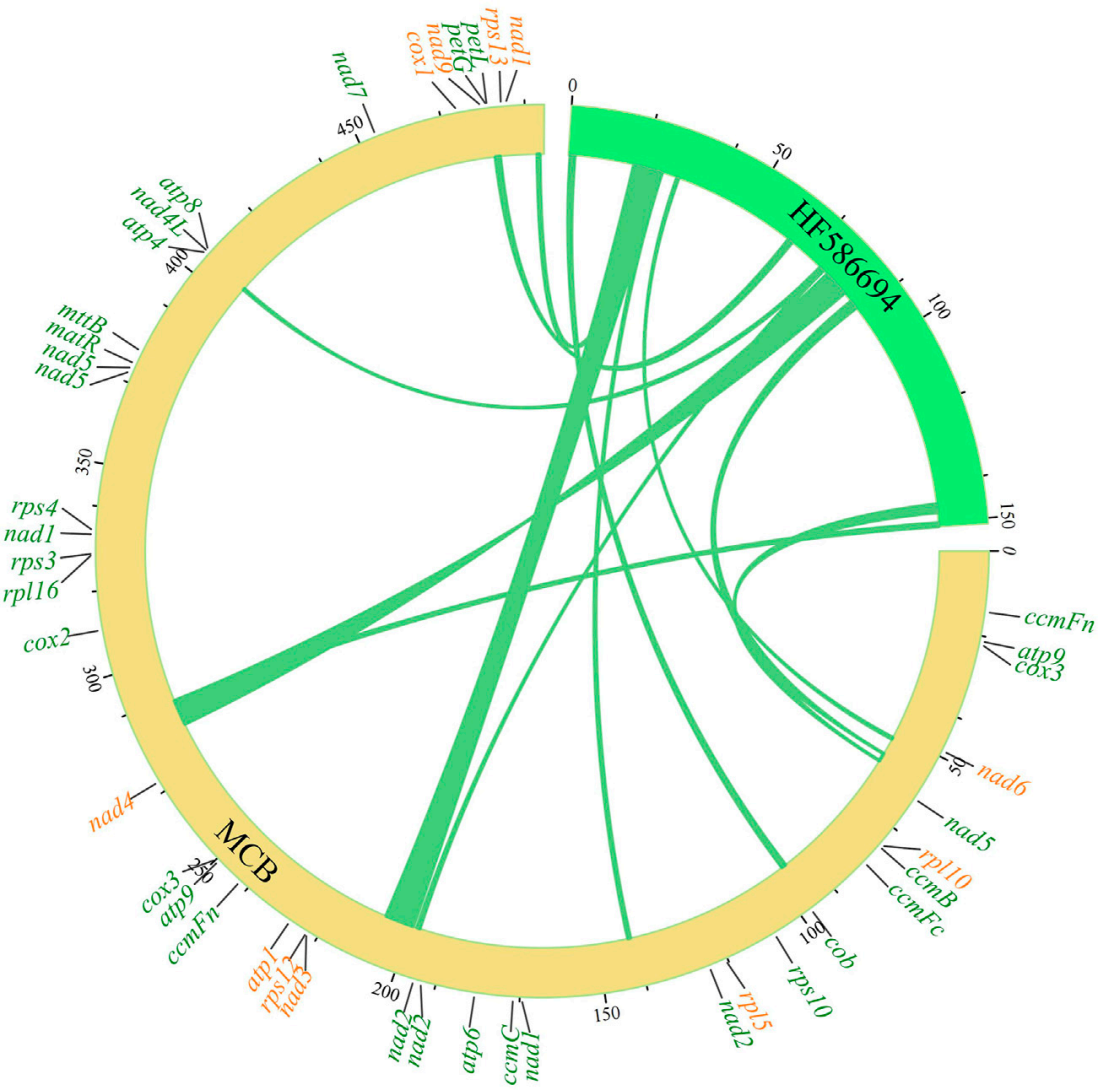
Gene transfer between the mitogenome and chloroplast genome of MCB.

In eukaryotic cells, genetic information transfer and biomass exchanges occurred between subcellular units or organelles (Schuldiner and Guo, 2015). Recently, studies showed that information exchanges and transfers between the mitochondria and chloroplasts existed in plant cells (Zhao et al., 2018). In this study, the phenomenon of gene exchange and the transfer was found between the chloroplast genome and mitogenome of MCB (Figure 2). In total, 11 segments (247,42 bp) transferred from the chloroplast genome to 12 mitochondrial segments (25,427 bp) were found with the 93%–97% sequence similarity, respectively (Supplementary Table S7). The longest and shortest transfer segments were 7,723 bp and 402 bp in length, respectively. Of these, there were two mitochondrial segments (141,923–141,239 bp and 492,819–493,503 bp) were transferred from the chloroplast segment (24,521–25,205 bp) with 97% sequence similarity.

### SSR and repeat sequence analysis

**FIGURE 3:**
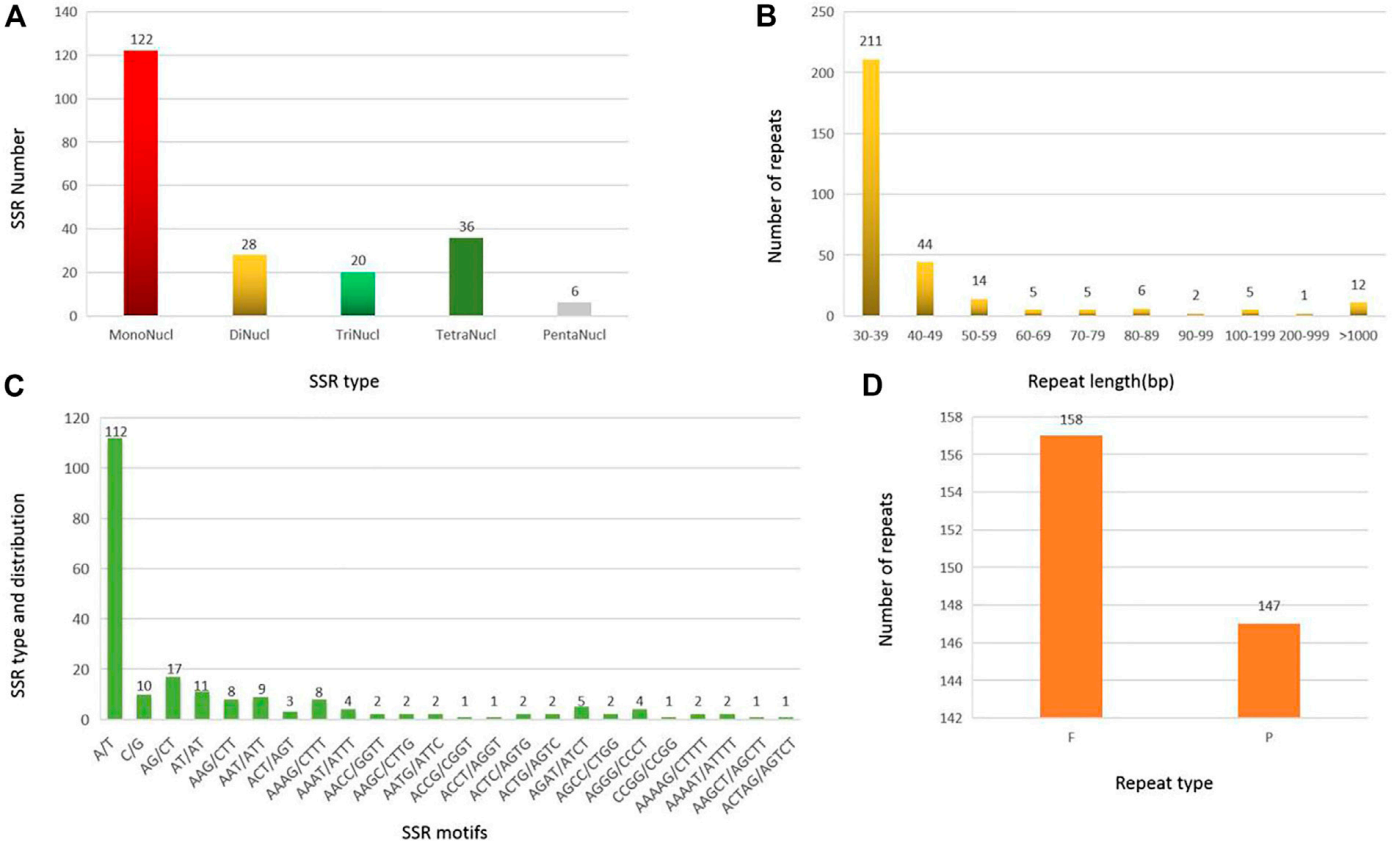
SSR and repeat analysis in MCB mitogenome. **(A)**, SSR number statistics of different SSR types; **(B)** Statistics of repeat sequence with different lengths; **(C)**, SSR motif analysis; **(D)**, Statistics of repeat sequence types.

A total of 212 SSRs were identified in the MCB mitogenome. Among them, more than half of the SSRs (122, 57.5%) belonged to monomers, while the dimers, trimers, tetramers, and pentamers were detected with lower frequency, accounting for 13.2%, 9.4%, 17.0%, and 2.8%, respectively (Figure 3A). Of the monomers, 112 A/T sequences (91.8%) occupied the main proportion, while G/C was only 10 (8.2%) (Figure 3C). Further, only six pentamers were observed, distributing in the IGS, *cob*, and, *matR* regions of the MCB mitogenome. The specific size and location of pentamers were shown in Supplementary Table S8.

**FIGURE 4:**
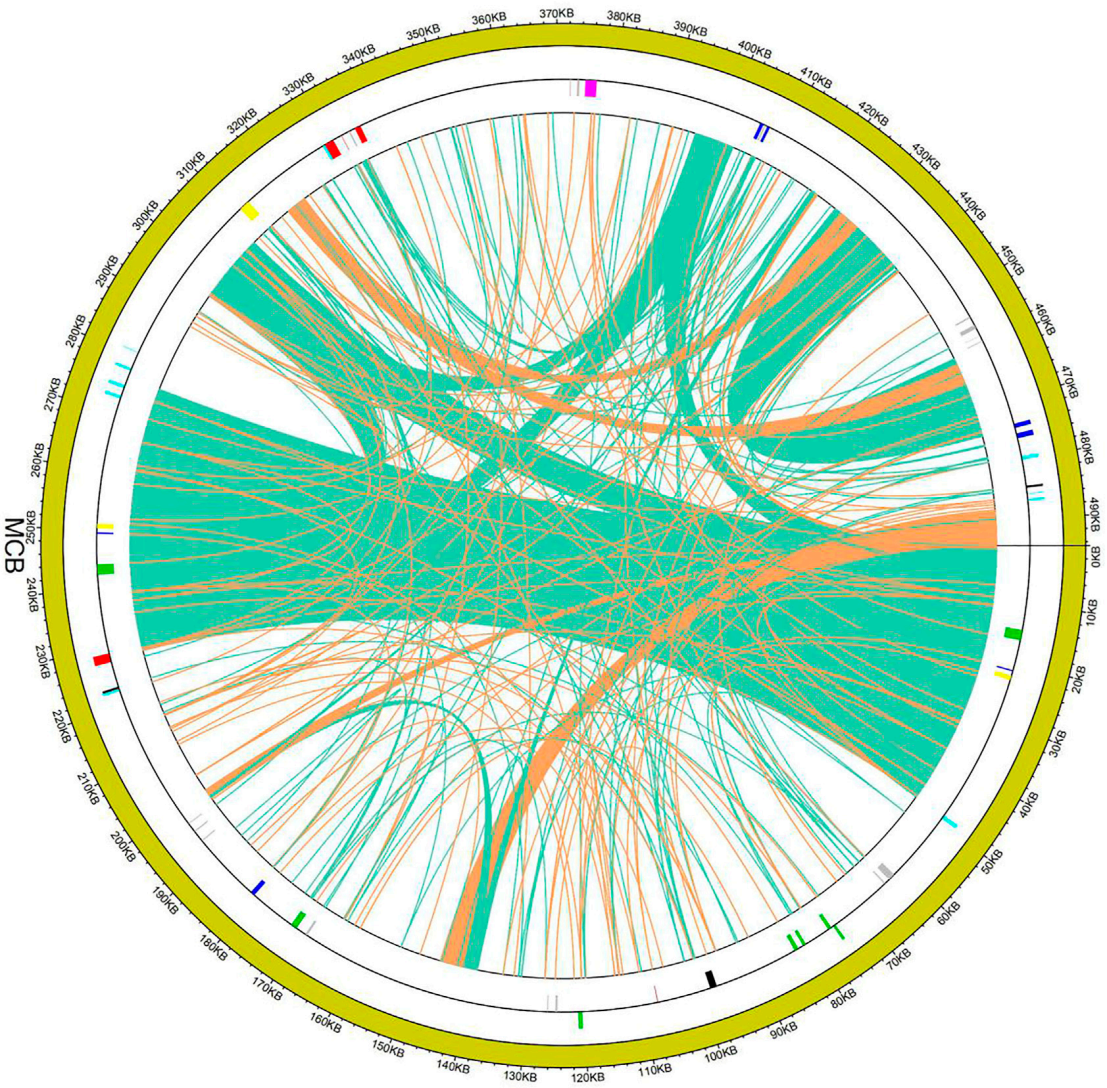
Distribution of repeat sequence in MCB mitogenome.

A total of 305 repeat sequences were identified in the MCB mitogenome with a total length of 139,861 bp and the length of each repeat ranged from 30 bp to 48,383 bp (Figure 4). As shown in Figure 3B, the 30–39 bp repeats were the majority type, accounting for 69.2% (211) of the total repeats. In addition, in terms of repeat types, there were 158 forward (F) (total length: 119,084 bp) and 147 palindromic (P) repeats (total length: 20,777 bp) in MCB mitogenome (Figure 3D).

The large repeat sequences (>1 kb) were notable because they were associated with reversible genomic structural changes (Bi et al., 2016). In this study, there were 12 repeats longer than 1 kb with a total length of 127,969 bp. Of these, 4 long palindromic repeats with a length of 15,475 bp and 8 long forward repeats with a length of 112,494 bp were detected, respectively (Supplementary Table S9).

### Collinearity analysis of the mitochondrial sequences

**FIGURE 5:**
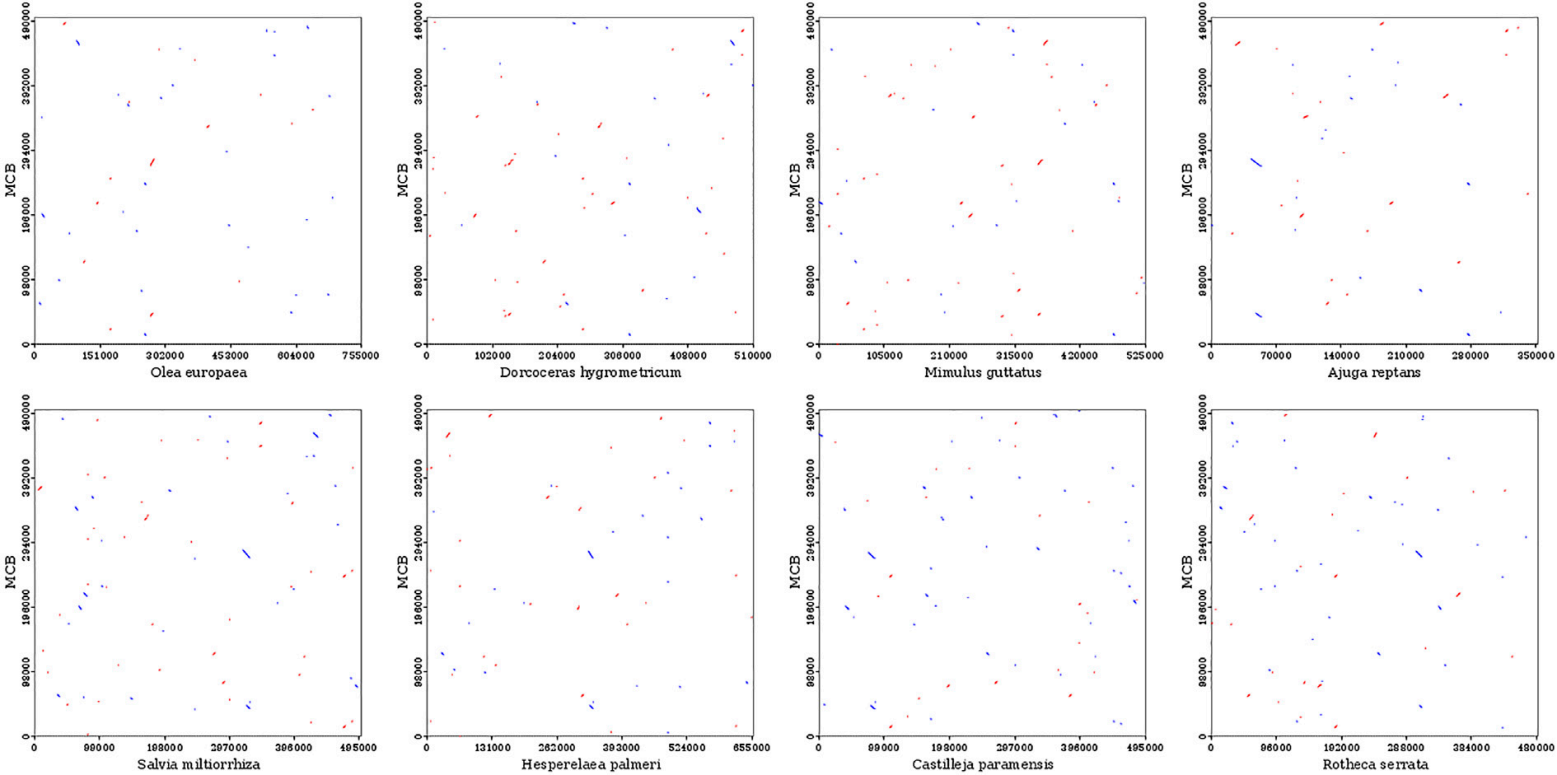
Dot-plot analysis of the mitochondrial sequences.

**FIGURE 6:**
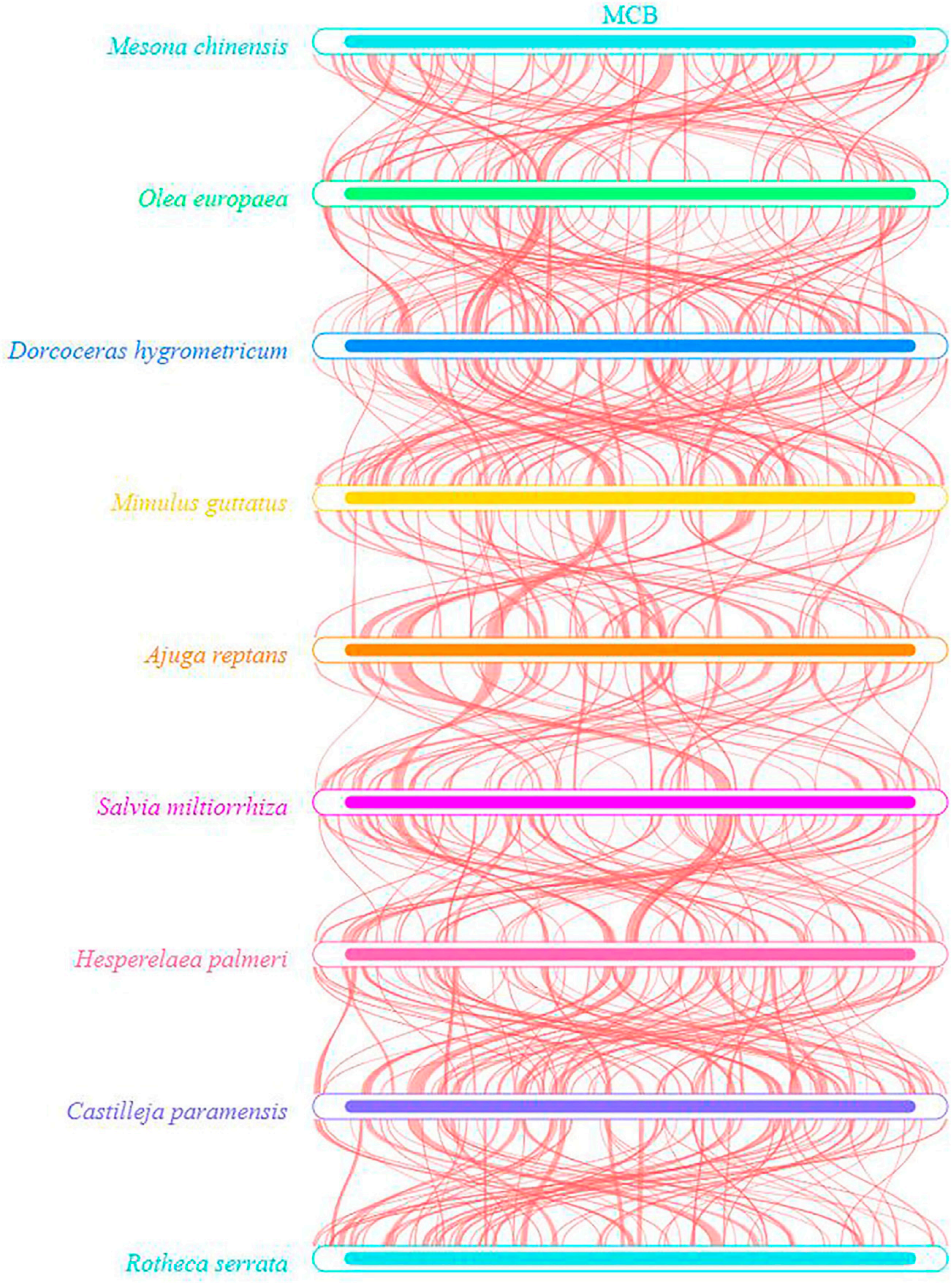
Collinearity analysis of the mitochondrial sequences.

To evaluate the degree of structural rearrangement between MCB and the same family species, the mitochondrial genome of MCB was compared with those of *Olea europaea (LR743801)*, *dorcoceras hygrometricum (NC_016741), Mimulus guttatus (NC_018041)*, *Ajuga reptans (NC_023103)*, *Salvia miltiorrhiza (NC_023209)*, *hesperelaea palmeri (NC_031323)*, *castilleja paramensis (NC_031806)*, *and rotheca serrata (NC_049064)*. As shown in Figure 5, when using MCB as a reference genome, the dot-plot analysis showed short stretches (less than 12 kb) of synteny across all species. In addition, there were many sequence rearrangements among these species, which indicated that the sequence arrangement order of the mitochondrial genome was not conservative. However, there were many homologous sequences among the species, especially the gene segments, which showed the conservative gene sequence of the mitochondrial genome (Figure 6).

### RSCU analysis of the MCB mitogenome

**FIGURE 7:**
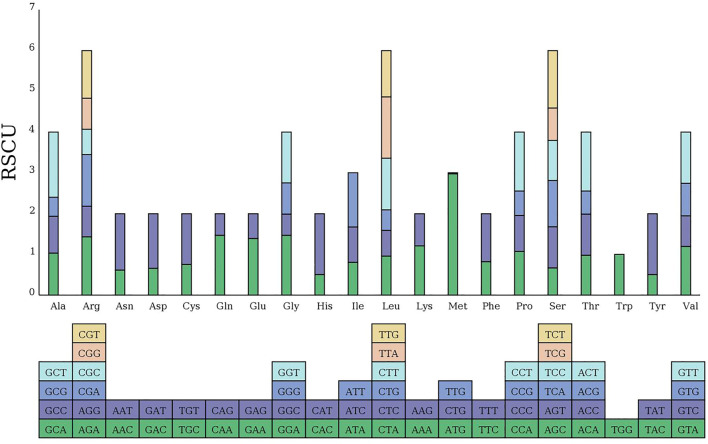
RSCU analysis of the MCB mitogenome.

The codon usage pattern of the MCB mitogenome was summarized (Figure 7). Overall, a total of 9,947 codons encoded the protein-coding genes in the MCB mitogenome. There were 64 different codons in the MCB mitogenome. Serine (909, 9.1%) and leucine (879, 8.8%) were the two most common amino acids, while terminator (32, .3%) was the least abundant amino acid in the MCB mitogenome. In addition, almost all the A/U-ending codons had RSCU values greater than one (RSCU > 1), while the C/G-ending codons possessed RSCU values less than one (RSCU < 1) (Supplementary Table S10).

### KaKs analysis

**FIGURE 8:**
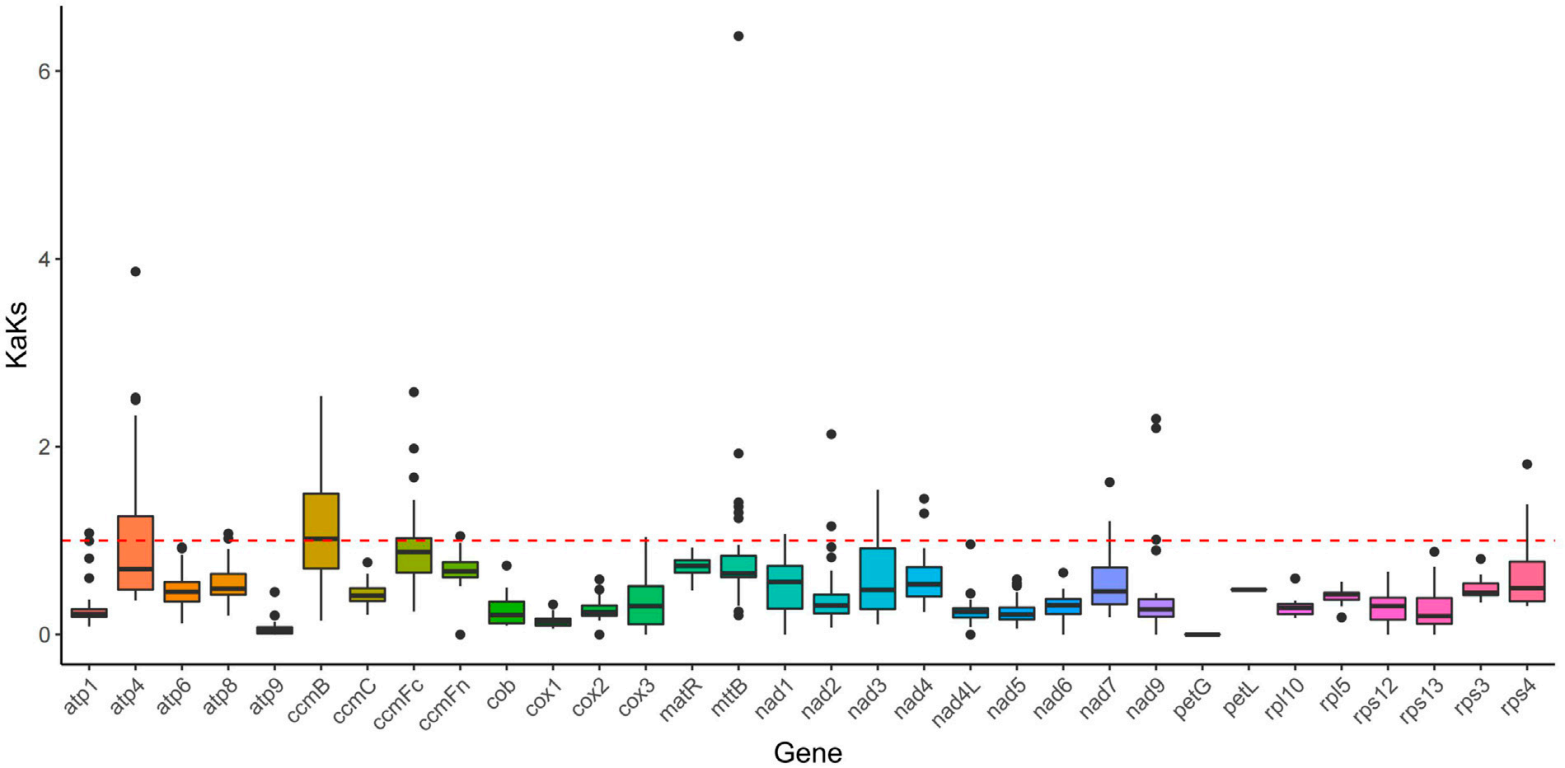
KaKs analysis of the MCB mitogenome.

In this study, a total of 32 genes were employed to compute the Ka/Ks ratio among the mitogenomes of *Mesona chinensis Benth*, *Olea europaea, dorcoceras hygrometricum*, *Mimulus guttatus*, *Ajuga reptans*, *Salvia miltiorrhiza, hesperelaea palmeri*, *castilleja paramensis*, *and rotheca serrata*. The results showed that almost all genes were subject to purification selection, except *ccmB*. Compared with other genes, *ccmB* might be subjected to greater positive selection pressure (Figure 8).

### Variation in the gene content and constitution in the Lamiaceae mitogenomes

**FIGURE 9:**
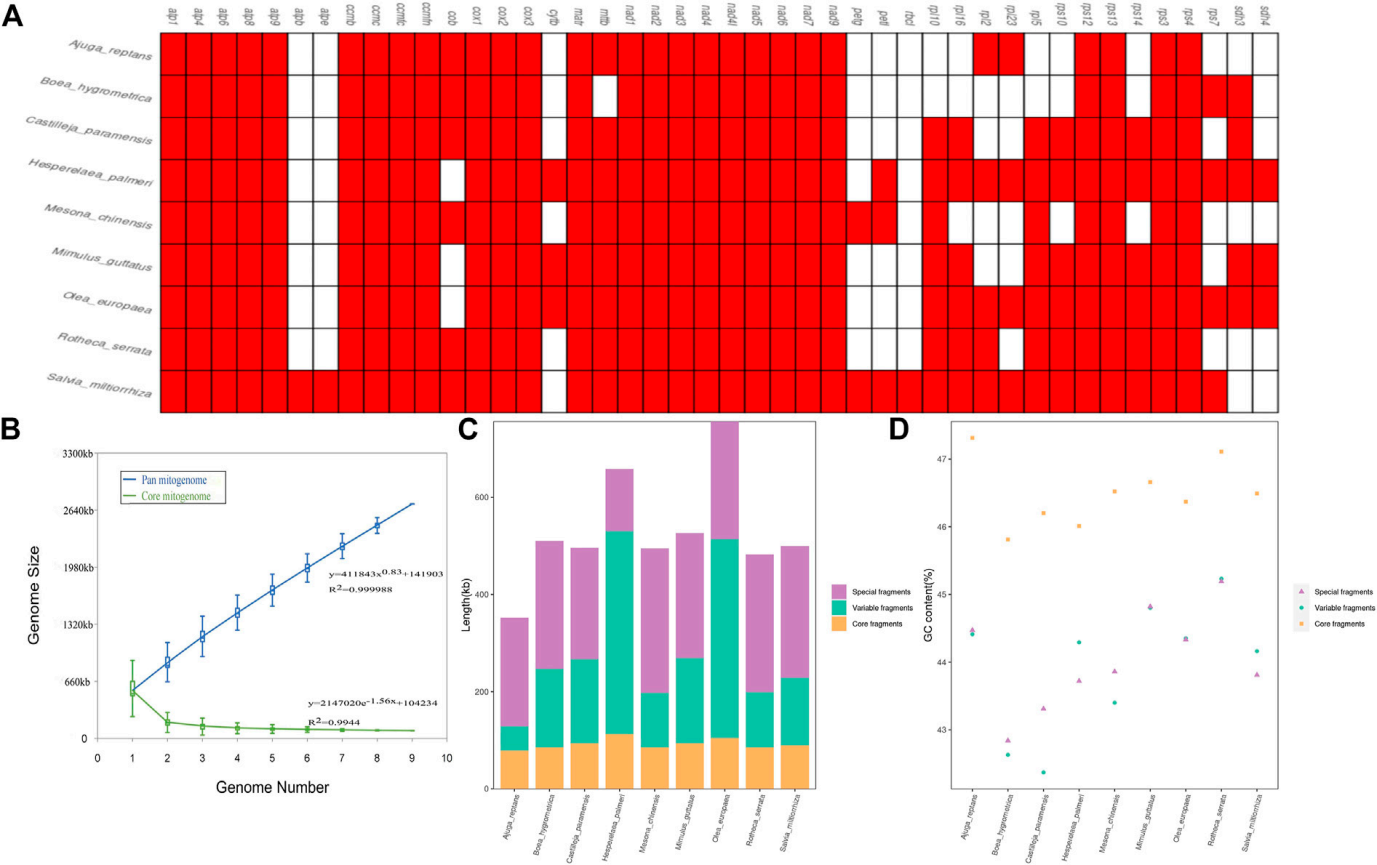
Variation in the gene content and constitution in Lamiaceae mitogenomes. **(A)**, The gene content of 9 Lamiaceae mitogenomes; **(B)**, Increase and decrease in the genome size of the pan mitogenome (blue) and core mitogenome (green); **(C)**, The size variation of different components in the 9 Lamiaceae mitogenomes; **(D)**, The GC content variation of different components in the 9 Lamiaceae mitogenomes.

In this study, we carried out a comparative analysis of mitochondrial gene content and genome size of the 9 Lamiaceae mitogenomes. The genome size variability of more than two-fold was observed among Lamiaceae mitogenomes with a range from 352,069 bp in *Ajuga reptans* to 755,572 bp in *Olea europaea* and the GC content of the 9 Lamiaceae mitogenomes ranged from 43.27% in *Boea hygrometrica* to 45.54% in *Rotheca serrata* (Supplementary Table S11). *atpB* and *atpE* were unique to the *Rotheca serrata* and *Salvia miltiorrhiza* mitogenomes and the *mttB* gene loss was unique to the *Boea hygrometrica* mitogenome (Figure 9A). Moreover, the analysis of pan-mitogenome and mitochondrial constitution showed that the pan-mitogenome curve indicated an open pan-mitogenome (Figure 9B). The specific, core and variable fragments have a large variation in size with ranges from 128,410 bp (∼19.50% of *Hesperelaea palmeri* mitogenome) to 296,682 bp (∼59.98% of *Mesona chinensis* mitogenome), 78,279 bp (∼22.23% of *Ajuga reptans* mitogenome) to 112,771 bp (∼17.12% of *Hesperelaea palmeri* mitogenome), and 49,949 bp (∼14.19% of *Ajuga reptans* mitogenome) to 417,418 bp (∼63.39% of *Hesperelaea palmeri* mitogenome), respectively (Supplementary Table S11; Figure 9C). These specific, core and variable fragments contained stable GC contents of 44.0%, 46.5%, and 44.0% on average, respectively (Supplementary Table S11; Figure 9D). The core fragments of the Lamiaceae mitogenomes harbored a higher GC content than the variable and specific fragments.

### Phylogenetic analysis

**FIGURE 10:**
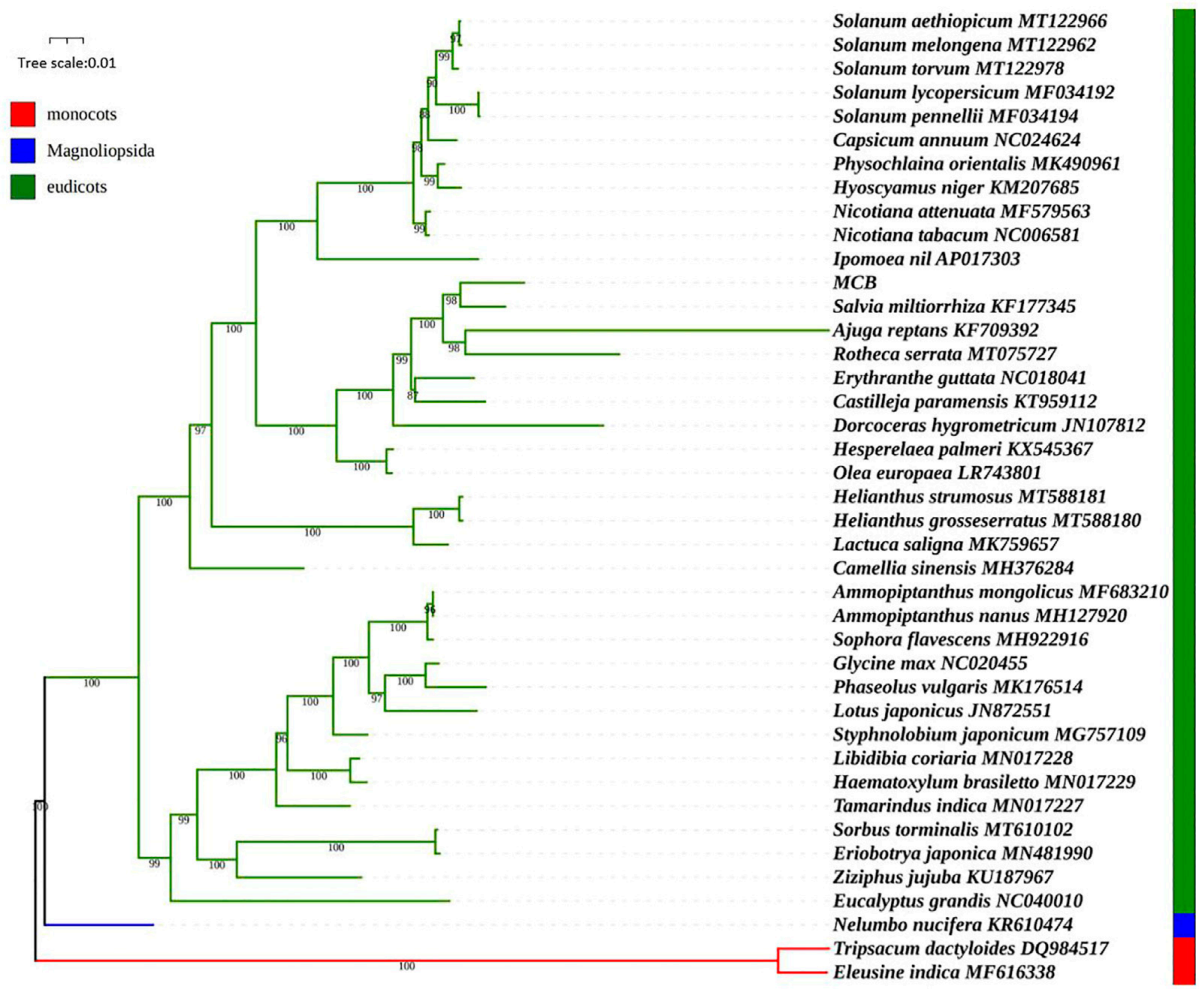
Phylogenetic analysis based on the mitogenoms of 41 species.

We used the maximum likelihood (ML) method to construct the phylogenetic tree based on the mitogenomes of 41 plant species. Of these, *Nelumbo nucifera* was magnoliopsida, *Eleusine indica* and *Tripsacum dactyloides* belonged to monocots, and the other 38 higher plants were eudicots (SupplementaryTable S1). As shown in Figure 10, the ML tree was divided into three clades, and the phylogenetic tree strongly supported the separation of the magnoliopsida, monocots, and eudicots plants. MCB belonged to eudicots Clade and stayed closest to *Salvia miltiorrhiza*. To further explore the utility of the mt genes in phylogenetic reconstruction, a total of 24 mt genes were selected for gene tree analysis. Results showed that many of the 24 gene trees were congruent with the previous reconstruction based on the mitogenomes, meanwhile, several gene trees had topologies inconsistent with the mitochondrial tree (Supplementary Figure S1). For example, in the *rps12* tree, *Salvia miltiorrhiza* was a sister to *Castilleja paramensis*, which was a sister of MCB in the mitochondria tree. And in the *nad7* tree, *Salvia miltiorrhiza* was a sister to *Rotheca serrata*, which was clustered as a sister to MCB in the mitochondria tree.

## Discussion

In the present study, we used the total genomic DNA to sequence and assembled the complete mitogenome of MCB based on Illumina and Nanopore sequencing data. This would lay a foundation for the whole genome analysis of MCB in the future. Generally, mitogenomes were described as single circular molecules (Kitazaki and Kubo 2010), while many other configurations of plant mitochondrial chromosomes had also been reported, such as highly branched and sigma-like morphologies, diverse linear and circular forms, and multichromosomal structures (Chen et al., 2017). Here, the mitogenome of MCB seemed to be a circular molecule (Figure 1). Circular arrangements appeared to be the common form among mitochondrial genomes assembled thus far in Lamiaceae (Qian, 2014; Zhu et al., 2014; Yu et al., 2022).

Plant mitochondrial genomes may vary enormously in size in different families and genera (Zhang et al., 2020), even within a single plant family (Chen et al., 2017). For example, in the Cucurbitaceae, mitogenomes varied over 7-fold in size, from 379 kb in *Citrullus lanatus* (Alverson et al., 2010) to 2,740 kb in *Cucumis melo* (Rodriguez-Moreno et al., 2011). In this study, as the members of the Lamiaceae family, the mitogenomes of *Ajuga reptans* (352,069 bp, NC_023103.1/KF709392.1), *Rotheca serrata* (482,114 bp, NC_049064.1/MT075727.1), *Salvia miltiorrhiza* (499,236 bp, NC_023209.1/KF177345.1), and *Mesona chinensis* Benth (494,599 bp) also varied enormously. The mitogenome of MCB was about 1.4 times that of *Ajuga reptans*, which supported the view that the mitogenome size was variable in angiosperm (Skippington et al., 2015). In addition, the mitogenome size of MCB was similar to that of *Salvia miltiorrhiza* and it might be due to their close genetic relationship.

Plant mitogenomes possessed a characteristic of their inclination to become a genetic “dumping ground” for sequences from both the chloroplast and nuclear genomes. Usually, sequence transfer from chloroplast to mitochondrial genomes typically was unidirectional (Tsunewaki, 2011; Chen et al., 2017). cpDNA-like sequences were detected in many mitogenomes from various species (Alverson et al., 2010; Rodriguez-Moreno et al., 2011). In the grape mitochondrial genome, there were 30 chloroplast fragments with a total length of 68,237 bp, accounting for 8.8% of the whole mitogenome and 42.4% of the whole chloroplast genome (Goremykin et al., 2009). Similarly, 17 chloroplast fragments with a total length of 22,593 bp and with a size range of 32–6,635 bp, account for 6.3% of the rice mitogenome (Notsu et al., 2002). In our investigation, 12 transfer segments were found with a total length of 22,075 bp (about 4.5% of the mitogenome) (Figure 2; Supplementary Table S7), supporting the conclusion that about .1%–10.3% of mtDNA originated from the chloroplast genome (Sloan and Wu, 2014).

Repetitive sequences including SSR, tandem repeats, short repeats, and large repeats were common in plant mitogenomes and about 38% of the mitogenome was obtained by the repeats of copy number and variable size (Mower et al., 2012). The presence of a large number of repeated sequences could promote the occurrence of mitochondrial sequence recombination, resulting in the generation of isomeric genomic sequences in mitochondria and sub-genomic sequences derived from some small circular molecular structures (Fauron et al., 1995). SSRs sequences were widely used in genetic analysis and species identification of individuals and populations due to their strong repeatability and relatively conserved characteristic (Choi and Park, 2015). SSRs were abundant in plants, however, the frequency of SSR in different plants varied greatly. In the mitogenome of *G. raimondii*, a total of 674 SSRs were identified and the single nucleotide SSR was the most (44.5%) (Bi et al., 2016). In this study, a total of 212 SSRs were detected in the MCB mitogenome, and 112 A/T sequences (52.8%) occupied the main proportion of the SSRs (Figure 3C). It was indicated that these mitochondrial SSRs could lay a foundation for the development of mitochondrial molecular markers, accurate identification and protection of genetic resources, and the evolution of flora in MCB.

Great variations in the genomic structure, gene content, and composition existed in the mitochondrial genomes of angiosperms (Mower et al., 2012). In this study, we could see that the size of the Lamiaceae mitogenomes was highly variable (Supplementary Table S11). The variation in mitogenome size could be explained by the differences in intergenic regions to a large extent (Mower et al., 2012) rather than the variable gene content (Figure 9A). Meanwhile, the differences in mitogenome size could also be attributed to the presence of foreign fragments and sizeable repetitive sequences (Park et al., 2015). Moreover, the relatively large mitogenomes (*Hesperelaea palmeri* and *Olea europaea*) always had larger variable fragments (*H. palmeri* 417,418 bp, 63.39% of the mitogenome and *O. Europaea* 409,030 bp, 54.14% of the mitogenome) (Supplemetary Table S11; Figure 9C). The differences in variable fragments could be explained by the genetic escape from the common ancestor of two or more species (Wang et al., 2018). In addition, the variable fragments showed relatively variable GC content and percent and low GC content among the Lamiaceae mitogenomes, indicating that these fragments might contain some foreign genetic materials with low GC content.

The characterization of the mitogenome of MCB stimulated a reassessment of the systematic relationships of MCB using the mt genomic/proteomic datasets. Given the demonstrated utility of mt proteomic datasets, high phylogenetic signal, and strong statistical support in trees (Jex et al., 2010; Park et al., 2011), there was a chance to test the phylogenetic relationships of MCB using the expanded mt datasets. In this study, the phylogenetic analysis showed that MCB stayed closest to *Salvia miltiorrhiza* (Figure 10). This result seemed to confirm the conclusion that the two genomes were similar in size.

## Conclusion

The MCB mitogenome was 494,599 bp in length and encoded 59 genes including 37 PCGs, 19 tRNAs, and 3 rRNAs. A total of 12 transfer segments and 212 SSR and 305 repeats were identified in the MCB mitogenome. In total, 9,947 codons were encoding the protein-coding genes in the MCB mitogenome. Serine and leucine were the two most abundant amino acids, while terminator was the least abundant amino acid. Almost all genes were subject to purification selection, except *ccmB*. *atpB* and *atpE* were unique to the *Rotheca serrata* and *Salvia miltiorrhiza* mitogenomes. *mttB* gene loss was unique to the *Boea hygrometrica* mitogenome. The core fragments of the Lamiaceae mitogenomes harbored a higher GC content than the variable and specific fragments. Phylogenetic analysis showed that MCB was closely related to *Salvia miltiorrhiza*. The current study provided valuable genomic resources for understanding and utilizing this important medicinal plant in the future.

## Data Availability

The MCB mitogenome sequence has been deposited in GenBank with accession numbers SRR21196489, SRR21196490, and OP537517.
