# Supplementary material for: Mitochondrial genome characteristics and phylogenetic analysis of the medicinal and edible plant Mesona chinensis Benth
Source: Front Genet. 2023 Jan 12;13:1056389. doi: 10.3389/fgene.2022.1056389 (PMC9878300; doi:10.3389/fgene.2022.1056389)
Supplement: Supplementary file 2 [file Table1.DOCX]

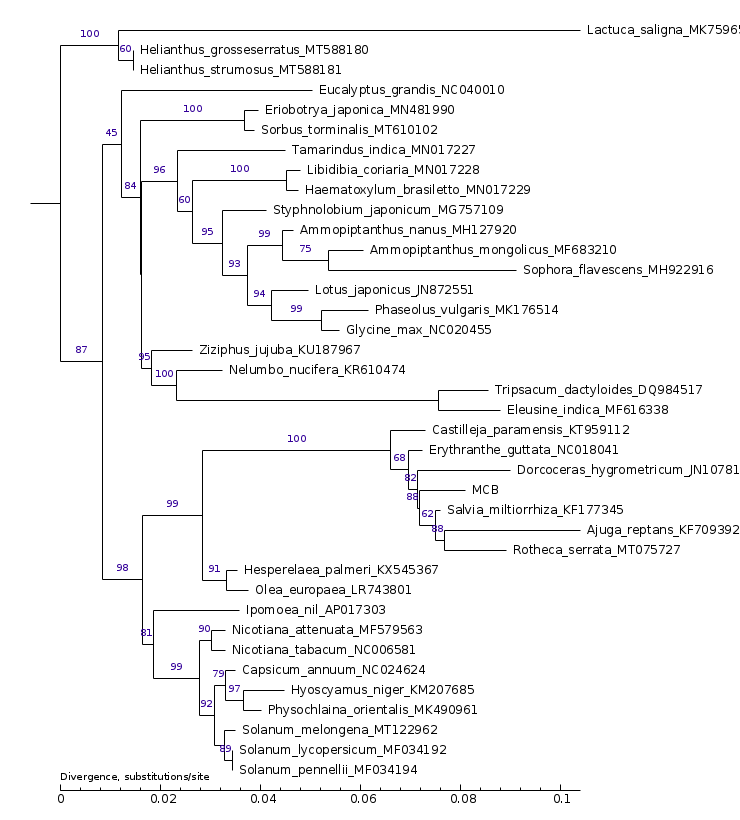


Figure S1. The maximum likelihood trees of 24 single mt genes (*atp1*).


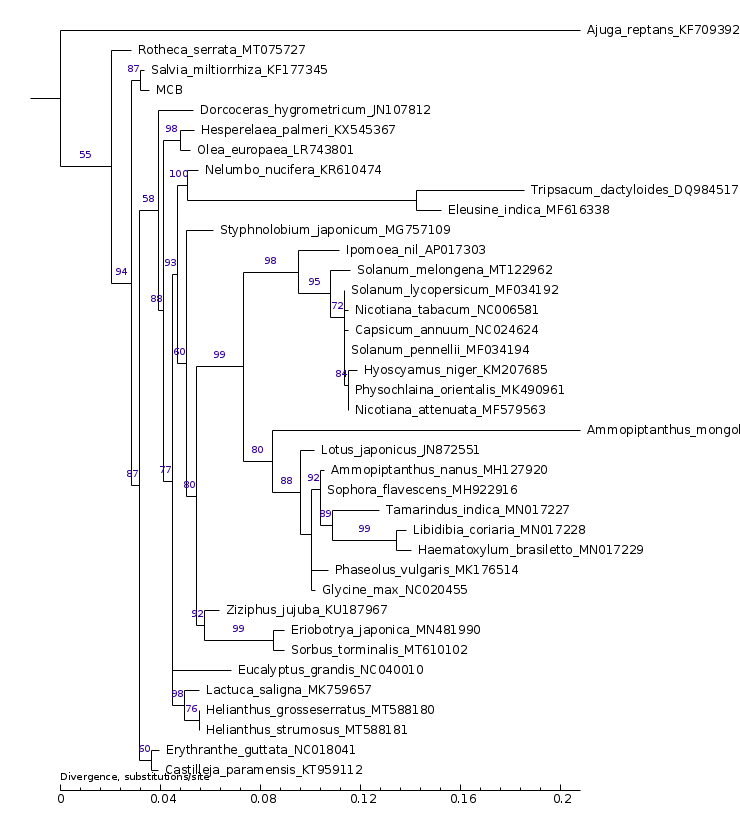


Figure S1. The maximum likelihood trees of 24 single mt genes (*atp4*).


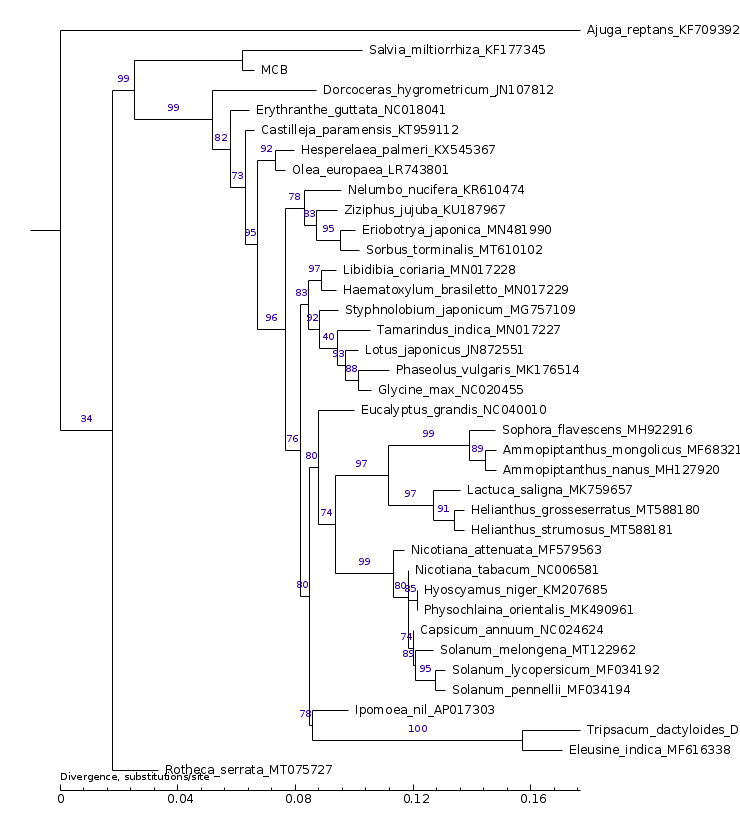


Figure S1. The maximum likelihood trees of 24 single mt genes (*atp6*).


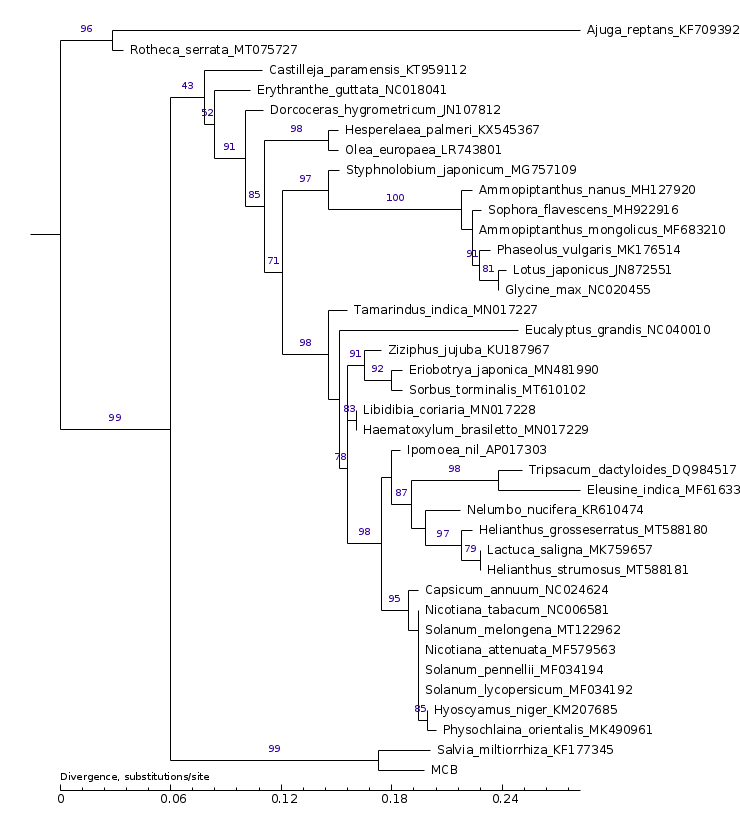


Figure S1. The maximum likelihood trees of 24 single mt genes (*atp9*).


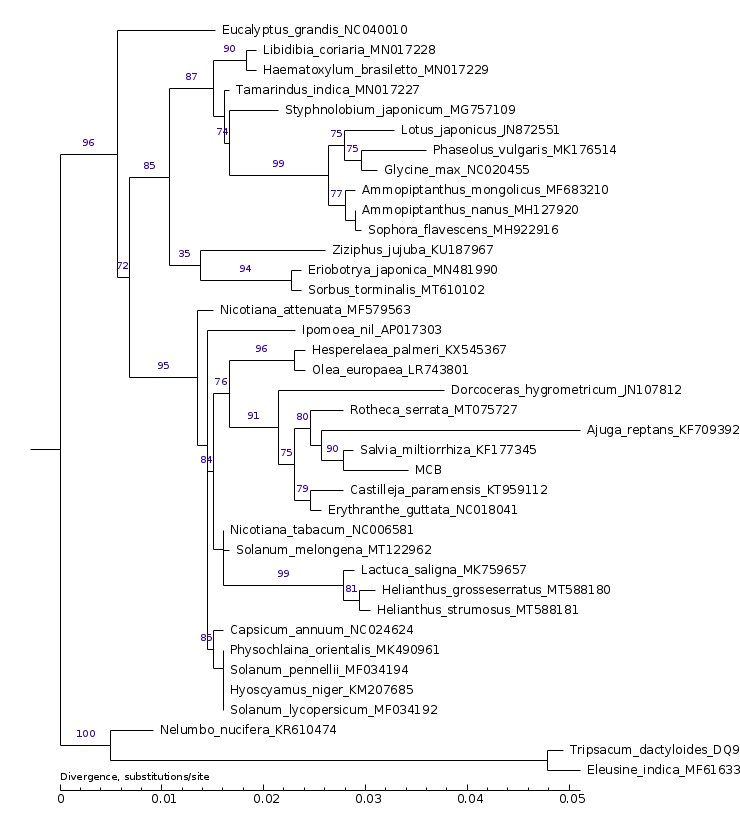


Figure S1. The maximum likelihood trees of 24 single mt genes (*ccmB*).


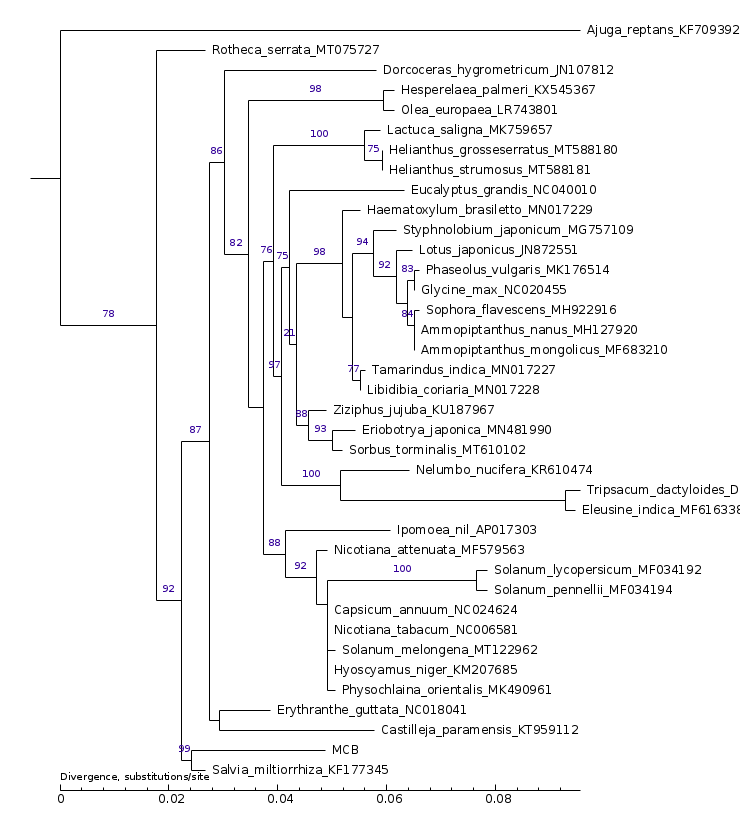


Figure S1. The maximum likelihood trees of 24 single mt genes (*ccmC*).


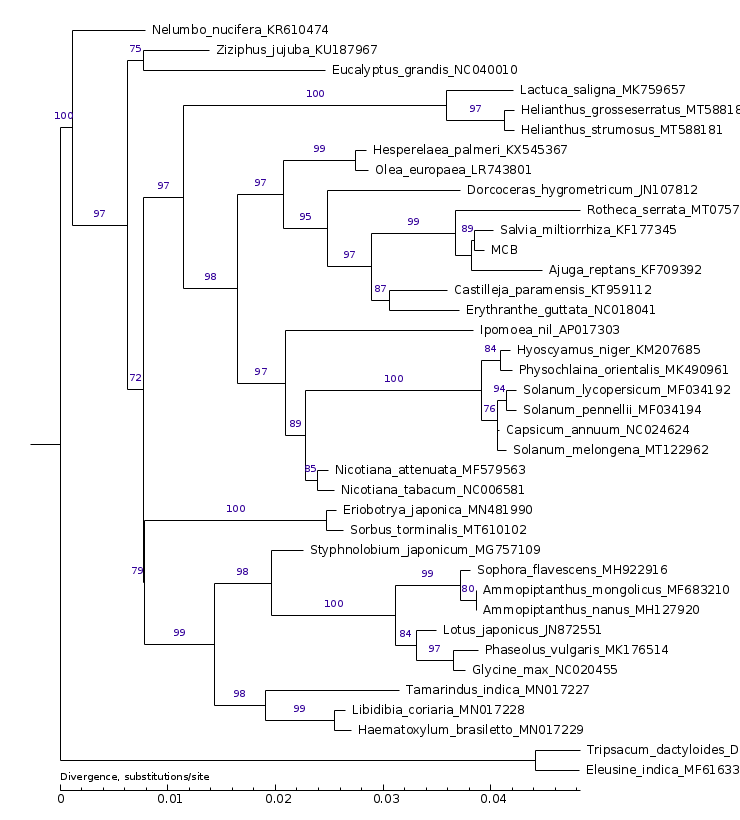


Figure S1. The maximum likelihood trees of 24 single mt genes (*ccmFn*).


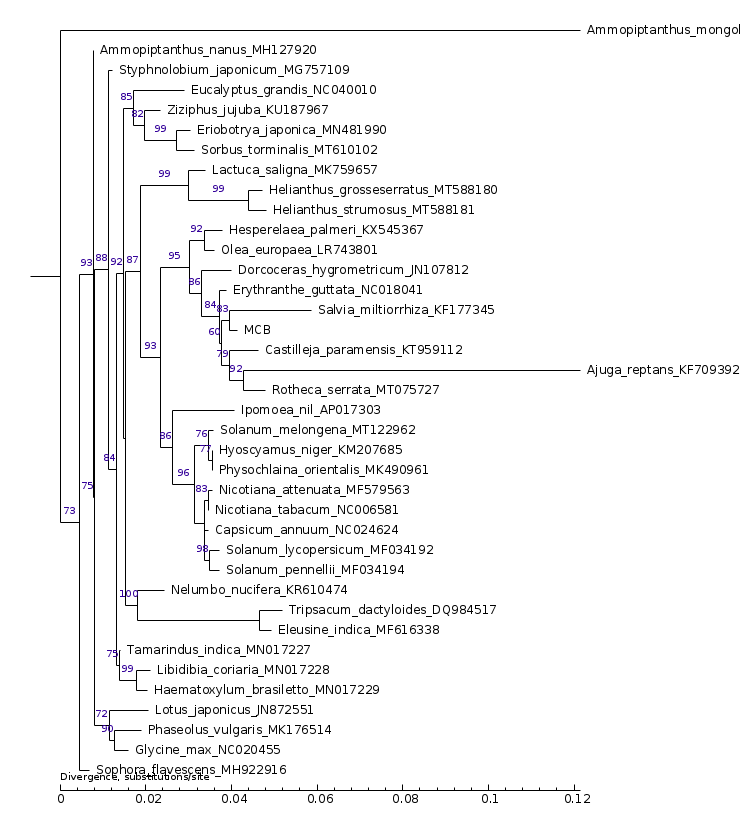


Figure S1. The maximum likelihood trees of 24 single mt genes (*cob*).


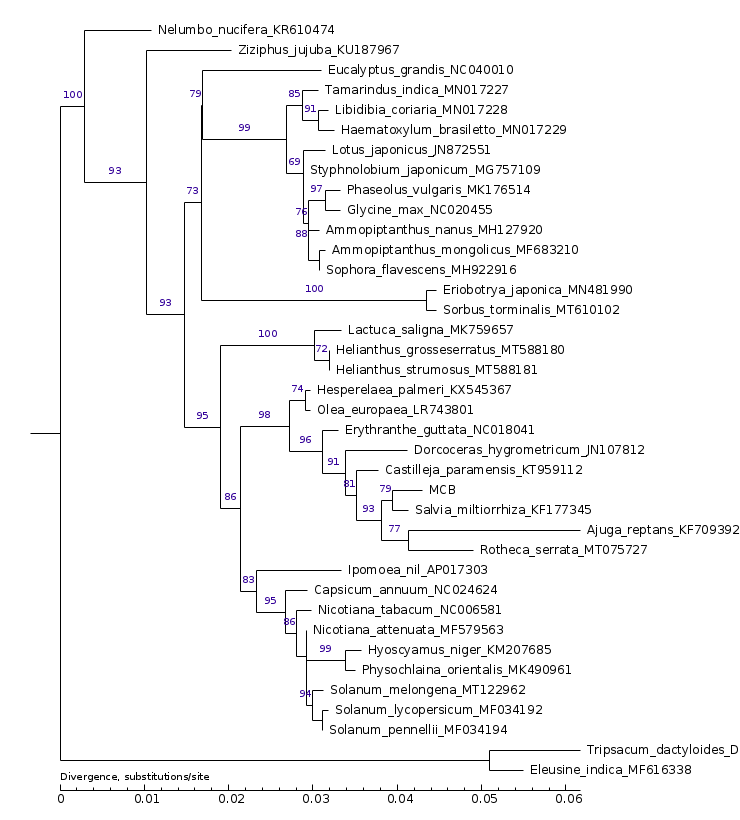


Figure S1. The maximum likelihood trees of 24 single mt genes (*cox1*).


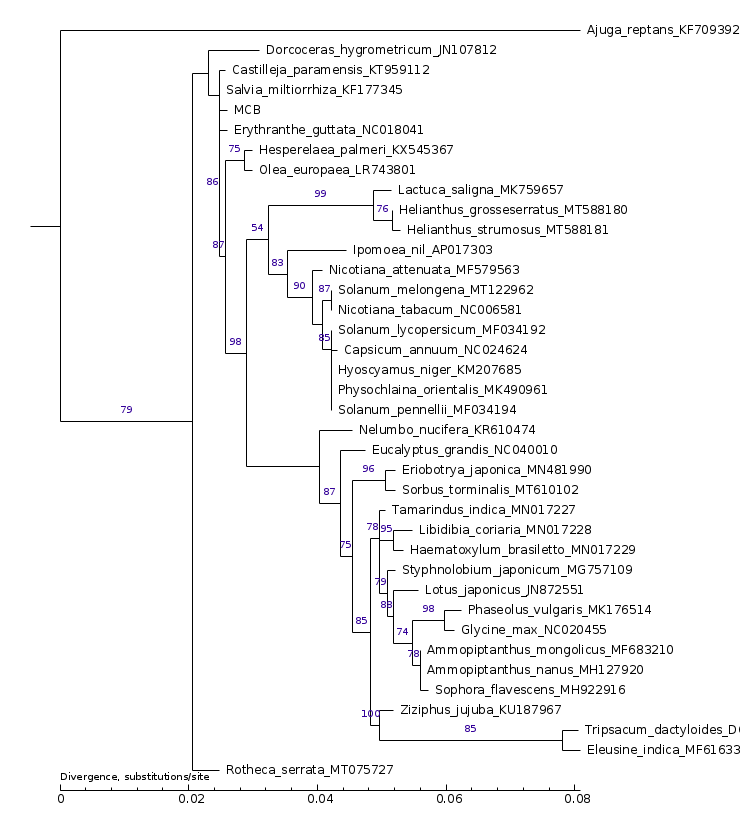


Figure S1. The maximum likelihood trees of 24 single mt genes (*cox3*).


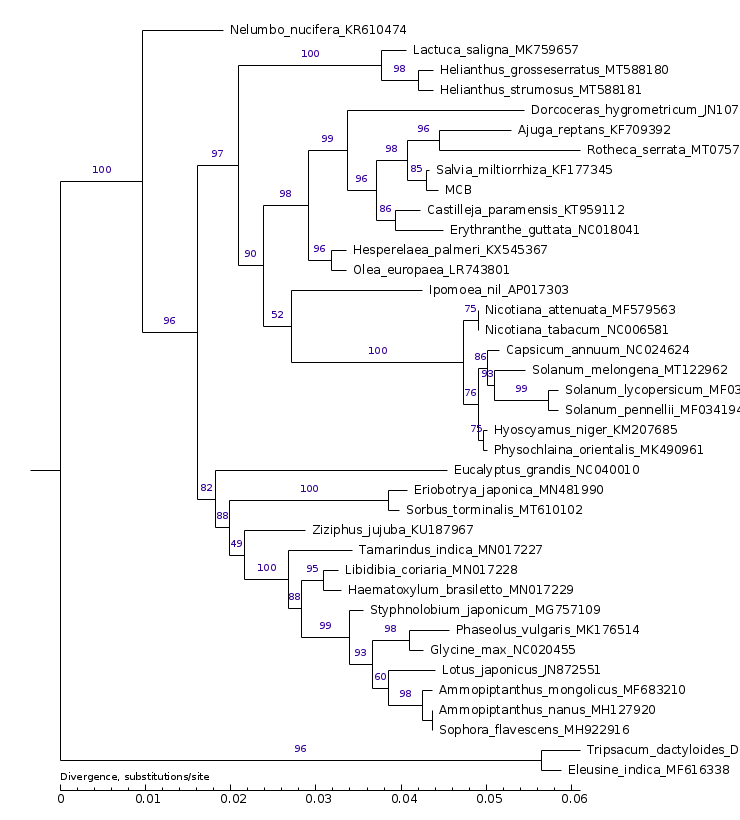


Figure S1. The maximum likelihood trees of 24 single mt genes (*matR*).


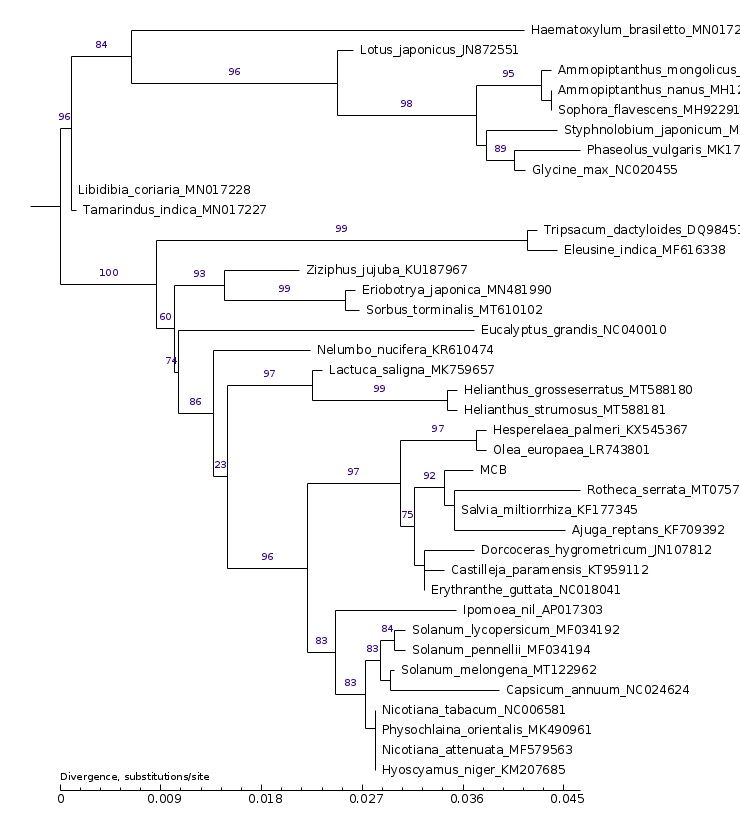


Figure S1. The maximum likelihood trees of 24 single mt genes (*mttB*).


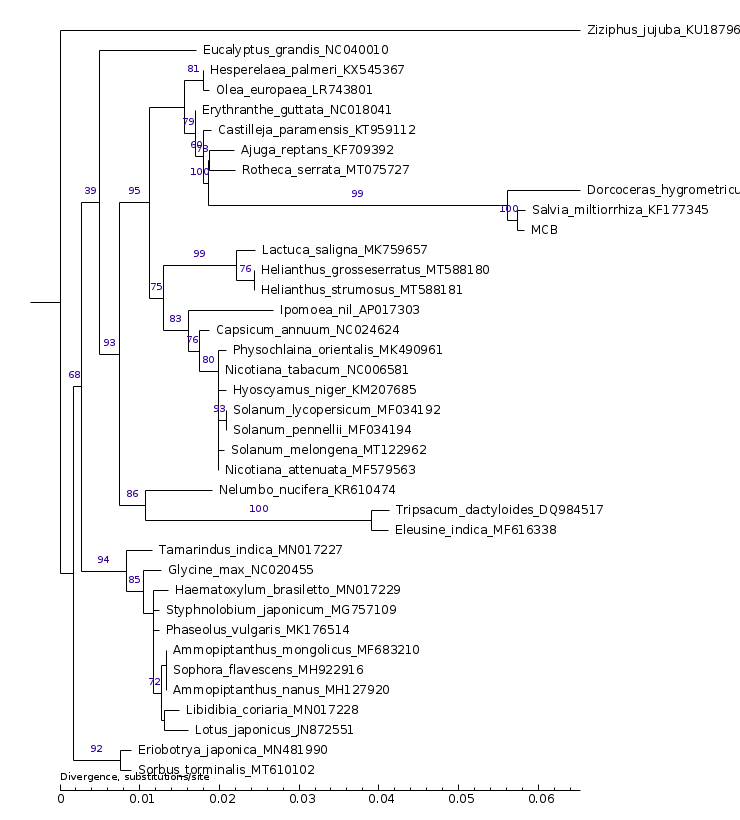


Figure S1. The maximum likelihood trees of 24 single mt genes (*nad1*).


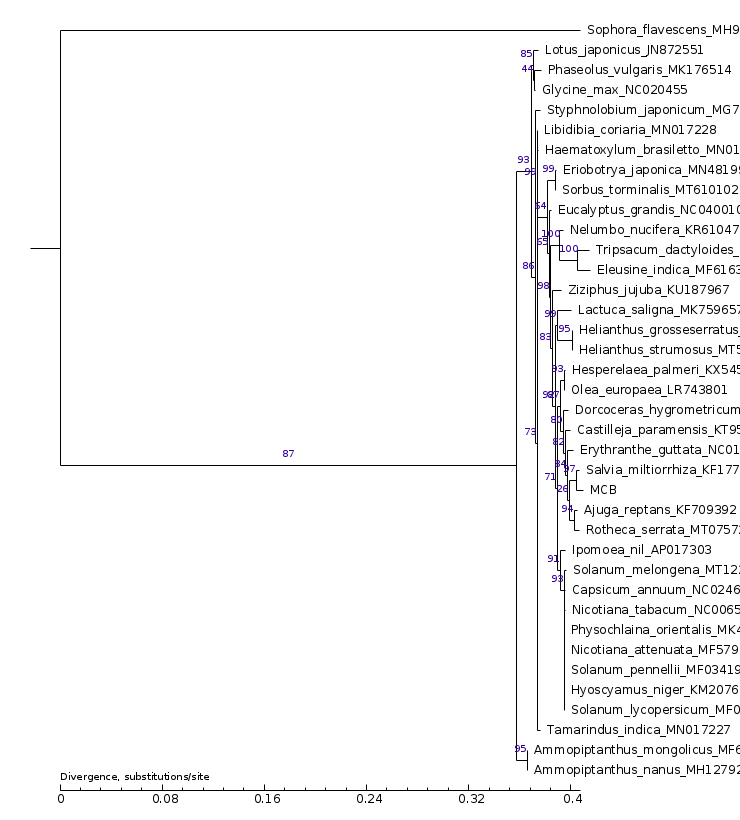


Figure S1. The maximum likelihood trees of 24 single mt genes (*nad2*).


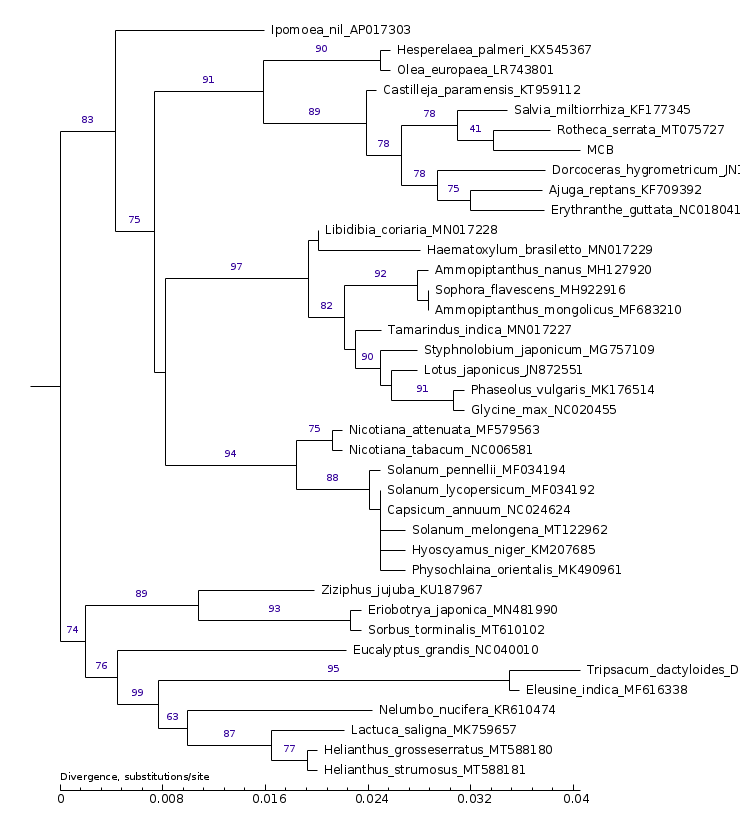


Figure S1. The maximum likelihood trees of 24 single mt genes (*nad3*).


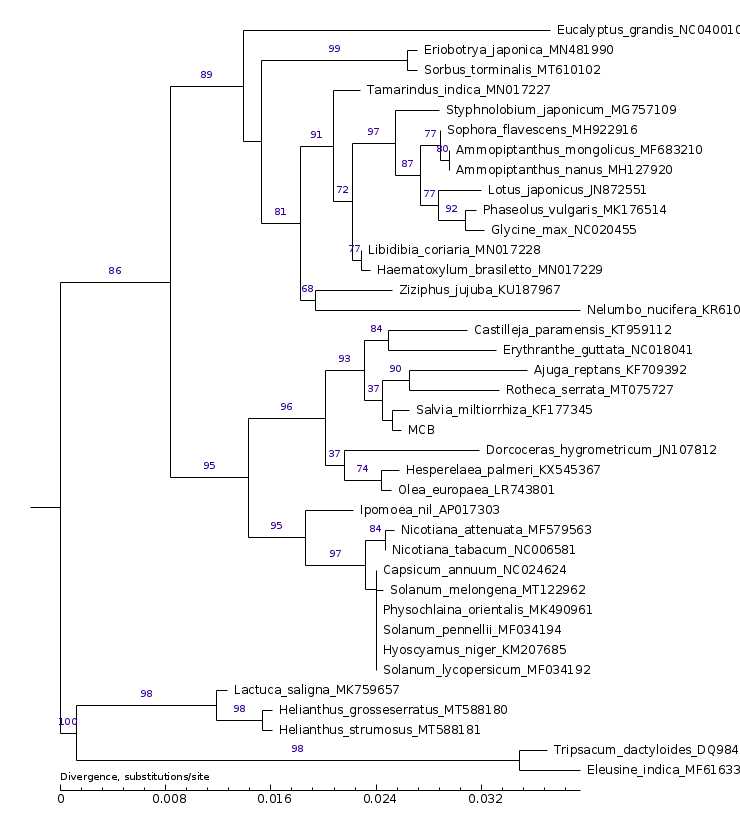


Figure S1. The maximum likelihood trees of 24 single mt genes (*nad4*).


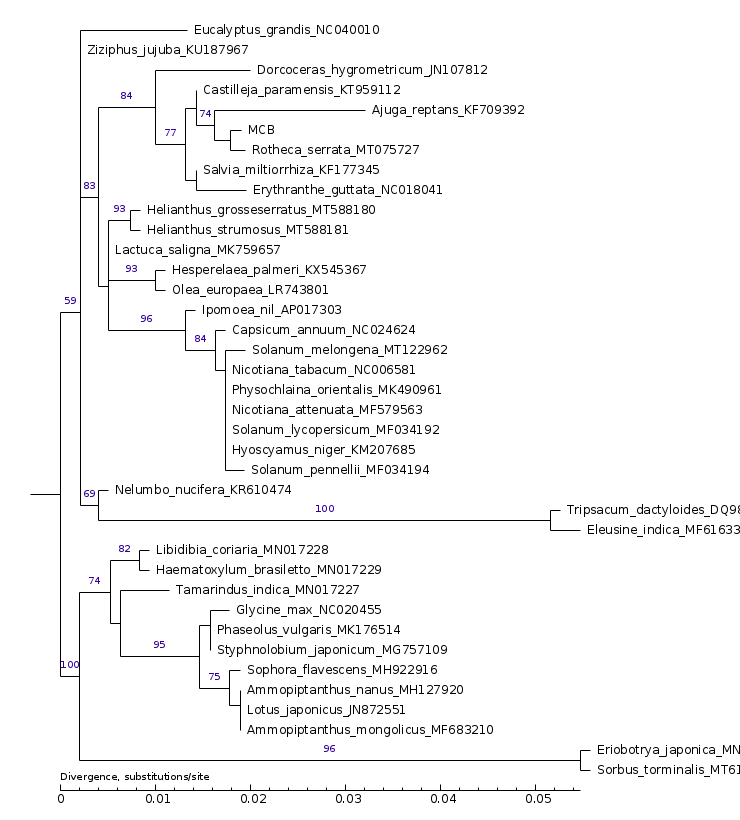


Figure S1. The maximum likelihood trees of 24 single mt genes (*nad4L*).


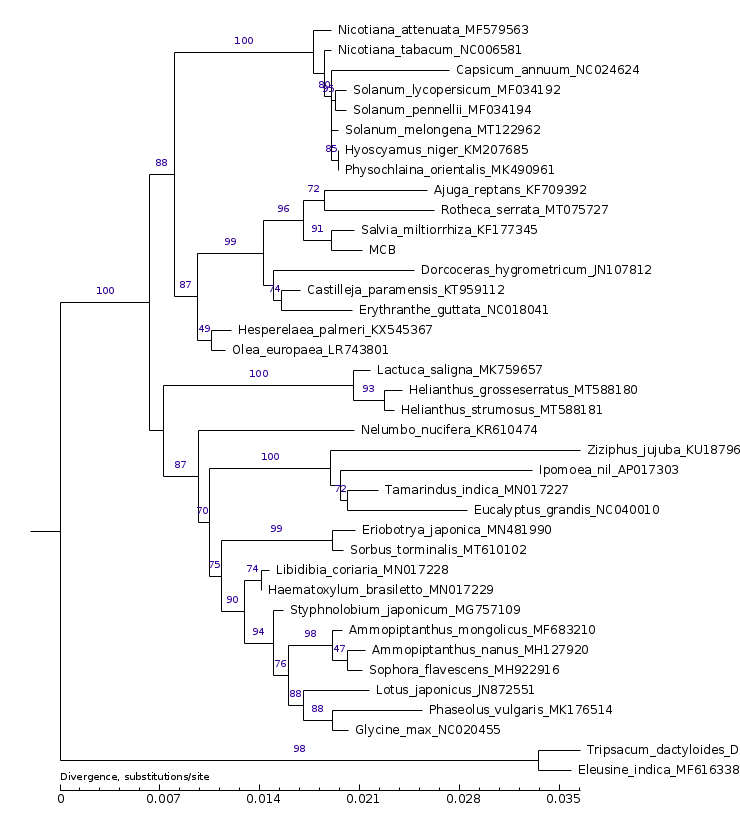


Figure S1. The maximum likelihood trees of 24 single mt genes (*nad5*).


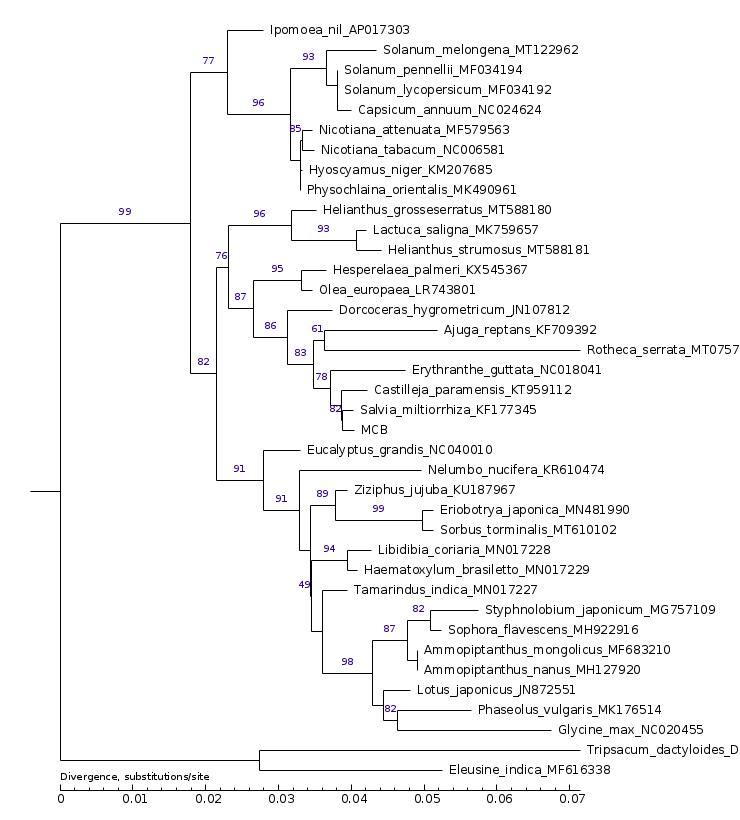


Figure S1. The maximum likelihood trees of 24 single mt genes (*nad6*).


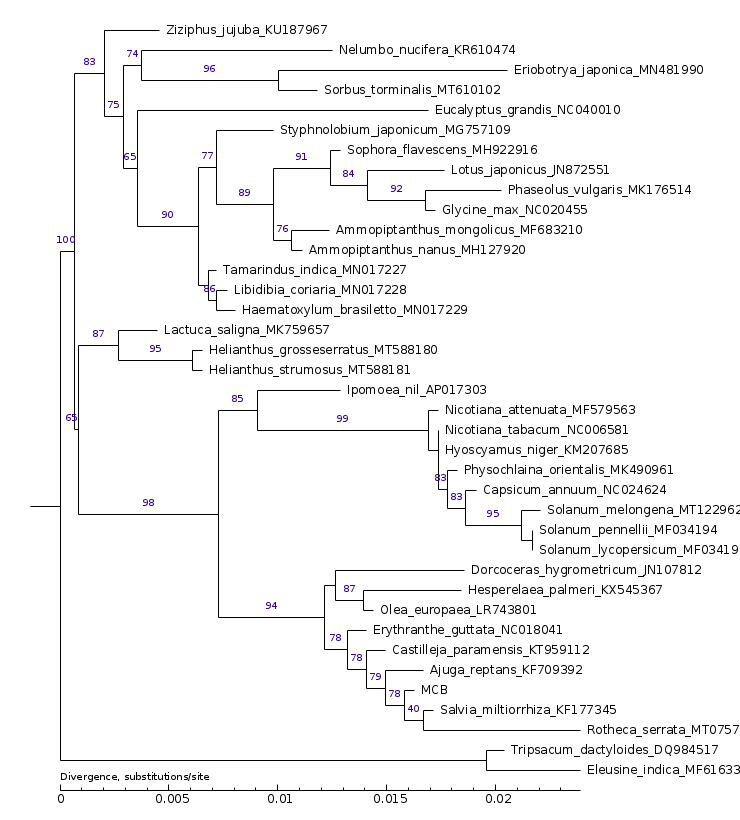


Figure S1. The maximum likelihood trees of 24 single mt genes (*nad7*).


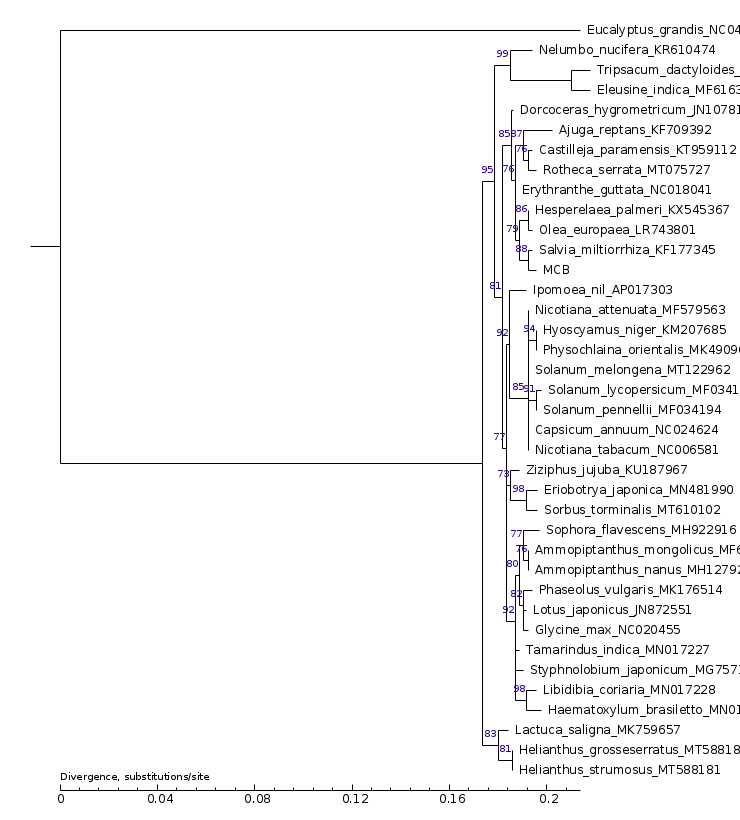


Figure S1. The maximum likelihood trees of 24 single mt genes (*nad9*).


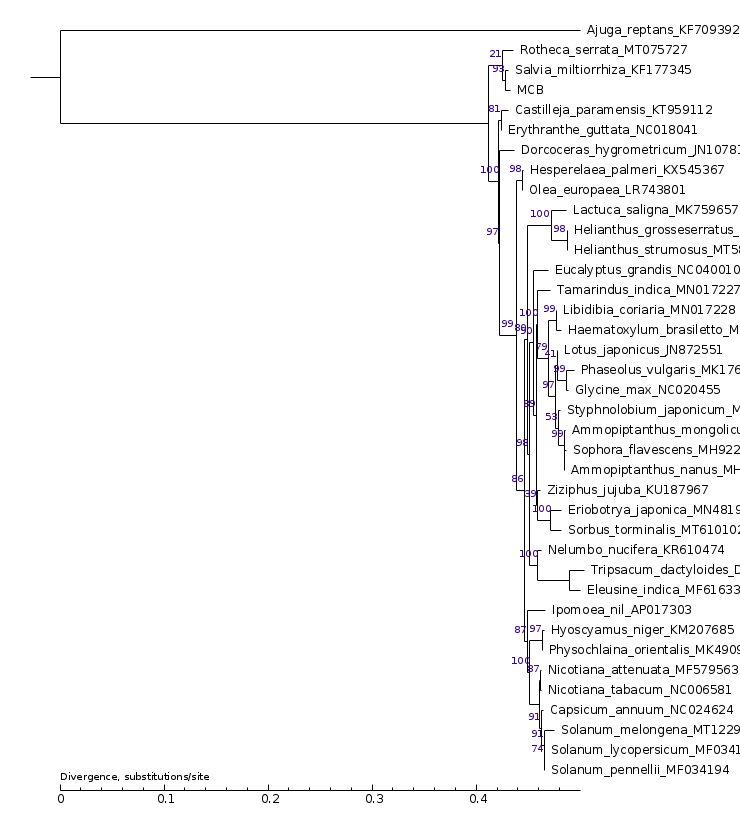


Figure S1. The maximum likelihood trees of 24 single mt genes (*rps3*).


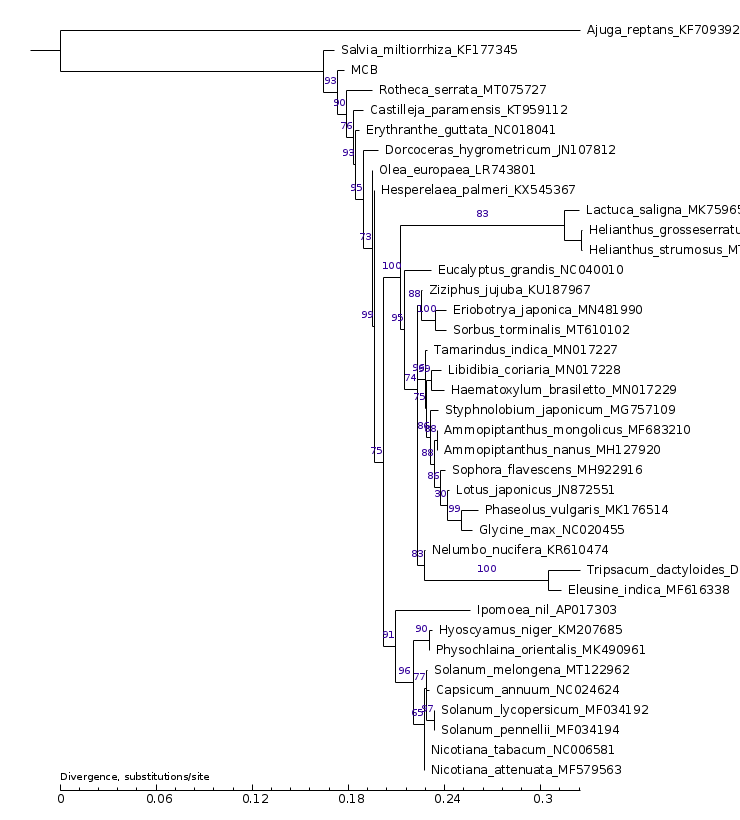


Figure S1. The maximum likelihood trees of 24 single mt genes (*rps4*).


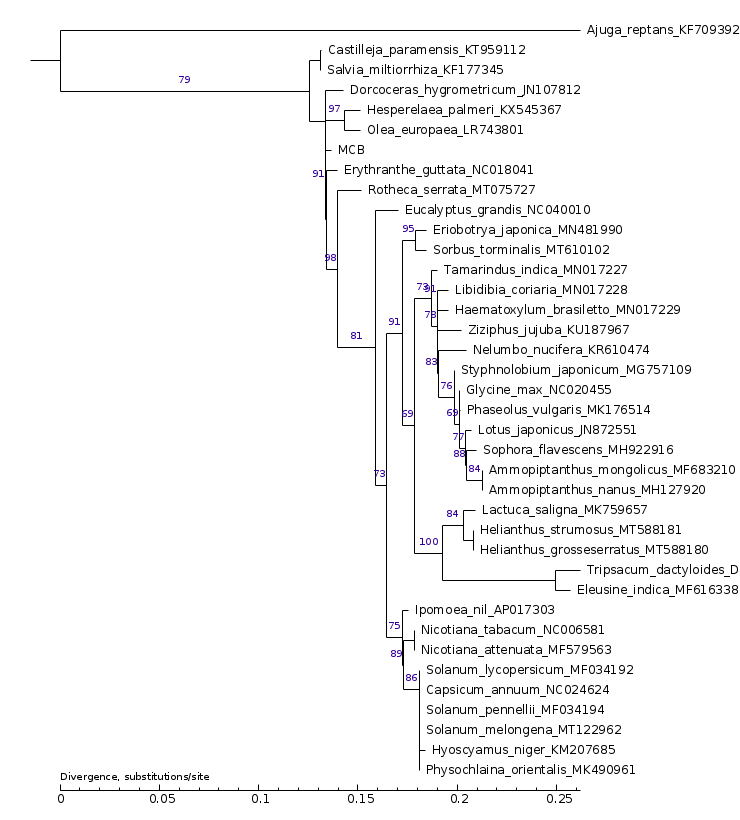


Figure S1. The maximum likelihood trees of 24 single mt genes (*rps12*).
